# Serum-integrated omics reveal the host response landscape for severe pediatric community-acquired pneumonia

**DOI:** 10.1186/s13054-023-04378-w

**Published:** 2023-03-01

**Authors:** Yi Wang, Xiaolan Huang, Fang Li, Xinbei Jia, Nan Jia, Jin Fu, Shuang Liu, Jin Zhang, Haiyan Ge, Siyuan Huang, Yi Hui, Chunrong Sun, Fei Xiao, Xiaodai Cui, Laurence Don Wai Luu, Dong Qu, Jieqiong Li, Jun Tai

**Affiliations:** 1 Experimental Research Center, Capital Institute of Pediatrics, Beijing, 100020 People’s Republic of China; 2 Department of Critical Medicine, Children’s Hospital Affiliated Capital Institute of Pediatrics, Beijing, 100020 People’s Republic of China; 3 Department of Otorhinolaryngology Head and Neck Surgery, Children’s Hospital Capital Institute of Pediatrics, Chinese Academy of Medical Sciences and Peking Union Medical College, Beijing, People’s Republic of China; 4 School of Life Sciences, University of Technology Sydney, Sydney, Australia; 5 Department of Respiratory and Critical Care Medicine, Beijing Institute of Respiratory Medicine, Beijing Chaoyang Hospital, Capital Medical University, Beijing, 100027 People’s Republic of China

**Keywords:** Community-acquired pneumonia, Proteomics, Metabolomics, Diagnosis, Host response

## Abstract

**Objective:**

Community-acquired pneumonia (CAP) is the primary cause of death for children under five years of age globally. Hence, it is essential to investigate new early biomarkers and potential mechanisms involved in disease severity.

**Methods:**

Proteomics combined with metabolomics was performed to identify biomarkers suitable for early diagnosis of severe CAP. In the training cohort, proteomics and metabolomics were performed on serum samples obtained from 20 severe CAPs (S-CAPs), 15 non-severe CAPs (NS-CAPs) and 15 healthy controls (CONs). In the verification cohort, selected biomarkers and their combinations were validated using ELISA and metabolomics in an independent cohort of 129 subjects. Finally, a combined proteomics and metabolomics analysis was performed to understand the major pathological features and reasons for severity of CAP.

**Results:**

The proteomic and metabolic signature was markedly different between S-CAPs, NS-CAPs and CONs. A new serum biomarker panel including 2 proteins [C-reactive protein (CRP), lipopolysaccharide (LBP)] and 3 metabolites [Fasciculol C, PE (14:0/16:1(19Z)), PS (20:0/22:6(4Z, 7Z, 10Z, 13Z, 16Z, 19Z))] was developed to identify CAP and to distinguish severe pneumonia. Pathway analysis of changes revealed activation of the cell death pathway, a dysregulated complement system, coagulation cascade and platelet function, and the inflammatory responses as contributors to tissue damage in children with CAP. Additionally, activation of glycolysis and higher levels of nucleotides led to imbalanced deoxyribonucleotide pools contributing to the development of severe CAP. Finally, dysregulated lipid metabolism was also identified as a potential pathological mechanism for severe progression of CAP.

**Conclusion:**

The integrated analysis of the proteome and metabolome might open up new ways in diagnosing and uncovering the complexity of severity of CAP.

**Supplementary Information:**

The online version contains supplementary material available at 10.1186/s13054-023-04378-w.

## Introduction

Community-acquired pneumonia (CAP) is the leading cause of death among children under five years of age globally, with 16.4 million hospitalizations every year [1, 2]. In China, a total of 1.42 million cases were reported as having one or more episodes of CAP, resulting in 1.48 million CAP episodes [3]. Approximately 8–20% of children hospitalized with CAP progress to severe disease, and many of these, especially infants, require admission to the pediatric intensive care unit (PICU) [1]. These severe cases require advanced interventions, such as invasive and non-invasive mechanical support to reduce the mortality rate of severe cases.

Diagnosis of pediatric CAP is often difficult due to the poor-quality evidence in clinical data, such as atypical imaging findings, complex clinical indicators, and poor prognostic signs [4, 5]. Failure to provide timely diagnosis and treatment may lead to acid-base balance disorders causing multiple organ failure and even septic shock in critically ill children. Thus, it is essential to develop new methods for early assessment of which cases are likely to become clinically severe. In addition, disease progression of CAP is a complex, multi-system process, and its underlying molecular mechanisms remain unclear. Changes in systemic responses may be caused by a complex set of factors including pathogens, genetic predisposition, and immune response. As a result, these factors may alter proteins and the downstream metabolites involved in disease progression [6]. Therefore, it is important to determine if host-derived proteins and metabolites in the circulation system are connected to the pathogenesis and progression of severe CAP.

Recent multi-omics studies have aimed to identify biomarkers and understand complex systemic changes which contribute to pathogenesis. Serum is the major container for small molecules whose relative amounts can provide valuable insights into disease pathogenesis [7, 8]. Previous studies have used serum proteins and/or metabolites to distinguish infectious disease from healthy controls. For example, one study identified a set of proteins able to accurately distinguish and predict COVID-19 outcomes [9], while in another study, metabolomics was combined with a random forest-based classification model and identified potential biomarkers for diagnosis of *Mycoplasma pneumoniae* pneumonia [10]. For CAP, metabolomics has been used to distinguish CAP from healthy individuals and identify metabolite signatures which correlate with disease severity [11]. Moreover, plasma lipidomics was also found to be useful in predicting the 90-day mortality prognosis in bacterial CAP [12]. Currently, in CAP, it is unclear which protein or metabolic pathways are involved in disease progression or what their combined roles are, especially in children. Thus, an integrated analysis of the proteome and metabolome may provide new avenues for understanding severe CAP.

Here, we used proteomics and metabolomics to profile the host response in CAP serum samples in a training cohort containing severe CAPs (S-CAPs), non-severe CAPs (NS-CAPs) and healthy controls (CONs). Our study uncovered several host proteins and metabolites that were altered in CAP. To identify potential biomarkers, we developed a machine learning-based pipeline that identified a combination of biomarkers that could accurately distinguish S-CAPs from controls. These selected biomarkers and combinations were then validated using enzyme-linked immunosorbent assay (ELISA) and metabolomics in a second validation cohort. Finally, the proteomics and metabolomics data generated in this study provided a global overview of the molecular changes, which may provide useful insight into the development of new therapeutics for treatment of CAP.

## Material and methods

### Ethical approval

The studies involving human participants were reviewed and approved by Ethical Committee of Capital Institute of Pediatrics (Ethical approval number: SHERLLM2019001). Written informed consent to participate in this study was provided by the participants’ legal guardian/next of kin.

### Patient enrollment

S-CAP patients were recruited from the PICU department in the Capital Institute of Pediatrics between 26th of December 2021 and 8th of March 2022. NS-CAP cases were enrolled from the respiratory department at the same time. CONs were collected from children who underwent a health checkup at the Capital Institute of Pediatrics. This study was approved by the Capital Institute of Pediatrics Ethics Committee.

Diagnosis of pediatric CAP was performed in accordance with the Chinese Medical Association guidelines as follows: younger than 18 years; symptoms started in communities; clinical signs of pneumonia (fever; tachypnea; increased respiratory work during examination; or auscultatory findings consistent with CAP); and pulmonary infiltration on the chest radiograph [13]. Severe cases required the following criteria: ICU treatment and positive pressure ventilation [14]. Among them, 1 patient had septic shock with the need for vasopressors. Characteristic and pathogenic types are supplied in Additional file 1.

### Evaluation of clinical characteristics and markers

Clinical information was retrospectively collected from the medical records of patients. This included proportion of blood cells [neutrophils (Neu), lymphocyte (Lym), monocytes (Mon)], white blood cells (WBC), procalcitonin (PCT), prothrombin time (PT), international normalized ratio (INR), activated partial thrombin time (APTT); fibrinogen (FIB), Fibrinogen degradation product (FDP) and thrombin time (TT). The non-invasive ventilation, invasive ventilation, days of hospitalization, ICU admission, and pediatric critical illness score (PCIS) were also assessed at hospital discharge.

### Proteomic analysis

Serum samples from cohort 1 were used for proteomics analysis (Additional file 1) as previously described [8, 15]. Briefly, each sample was lysed with 100 μL lysis buffer (8M urea in 100 mM triethylammonium bicarbonate, TEAB) at 25 °C for 30 min. The lysates were reduced by 5 mM Tris (2-carboxyethyl) phosphine (Pierce, Rockford, IL, USA) and incubated at 37 °C for 30 min with shaking (300 rpm). Next, samples were alkylated by 15 mM Iodoacetamide (Sigma-Aldrich, St. Louis, MO, USA) and digested with trypsin overnight at 37 °C. Then, mass spectrometry-grade trypsin gold (Promega, Madison, WI, USA) was used with an enzyme-to-protein ratio of 1:50. The dried peptides were dissolved in 20 μL loading buffer (1% formic acid, FA; 1% acetonitrile, ACN). Ten μL of sample was applied for LC–MS/MS analysis on an Orbitrap Fusion Lumos in data-dependent acquisition (DDA) mode coupled with Ultimate 3000 (Thermo Fisher Scientific, Waltham, MA, USA). The samples were loaded and separated by a C18 trap column (3 mm 0.10 × 20 mm).

For MS detection, the following parameters were used: full MS survey scans were performed in the ultra-high-field Orbitrap analyzer at a resolution of 120,000 and trap size of 500,000 ions over a mass range from 300 to 1400 m/z. MS/MS scan were detected in IonTrap and the 20 most intense peptide ions with charge states 2 to 7 were subjected to fragmentation via higher energy collision-induced dissociation (5 × 10^3^ AGC target, 35 ms maximum ion time). The resultant mass spectrometry data were analyzed using Maxquant (Version 2.1.0.0) and the protein search database used was the *Homo sapiens* FASTA database downloaded from UniprotKB (UP000005640.fasta). The following search parameters were used for Maxquant: precursor ion mass tolerance was set at 20 ppm; full cleavage by trypsin was selected; a maximum of two missed cleavages was allowed; static modifications were set to carbamidomethylation of cysteine, and variable modifications were set to oxidation of methionine and acetylation of peptides’ N-termini. The remaining parameters followed the default Maxquant setup. For protein identification, the following criteria was used: (1) peptide length ≥ 6 amino acids; (2) FDR ≤ 1% at the PSM, peptide and protein levels. Peptides were quantified using the peak area derived from their MS1 intensity and analyzed by perseus.

### Enzyme-linked immunosorbent assay (ELISA)

ELISA was used to quantify the concentrations of selected serum proteins. Samples from cohort 2 were used for ELISA verification. Adiponectin (ADIPOQ), apolipoprotein C (APOC1), vitamin K-dependent protein C (PROC), angiotensinogen (AGT), fibronectin (FN1), histidine-rich glycoprotein (HRG), albumin (ALB), C-reactive protein (CRP), and lipopolysaccharide (LBP) ELISA kits (Inselisa) were used to measure the proteins changes in serum from participants in the training (cohort 1) and testing (cohort 2) datasets. ELISAs were performed according to each kit’s instructions.

### Metabolomic analysis

All serum samples (Additional file 1) were used for metabolomics analysis as described previously [8, 15]. Quality control (QC) samples were applied by mixing equal amounts of all samples to ensure data quality for metabolic profiling. Samples (100 μL) were extracted by 400 μL of MeOH/ACN (1:1, v/v) solvent mixture, and then incubated and centrifuged for 10 min at 13,500 g at 4 °C. Next, the supernatant was divided into three fractions: two for reverse-phase/ultra-performance liquid chromatography (RP/UPLC)-MS/MS methods with positive ion-mode electrospray ionization (ESI) and negative-ion mode ESI, and one for hydrophilic interaction liquid chromatography (HILIC)/UPLC-MS/MS with positive-ion mode ESI.

All UPLC-MS/MS methods used the ACQUITY 2D UPLC system (Waters, Milford, MA, USA) and Q-Exactive Quadrupole-Orbitrap (QE, Thermo Fisher Scientific™, San Jose, USA) and TripleTOF 5600 + (AB SCIEX, MA, USA) with ESI source and mass analyzer. In the UPLC-MS/MS method, the QE was operated under ESI coupled with a C18 column (UPLC BEH C18, 2.1 × 100 mm, 1.7 μm; Waters). The mobile solutions used in the gradient elution were water and methanol containing 0.1% FA. When the QE was operated under negative ESI mode, the UPLC method used a C18 column eluted with mobile solutions containing methanol and water in 6.5 mM ammonium bicarbonate at pH 8. The UPLC column used in the hydrophilic interaction method was a HILIC column (UPLC BEH Amide, 2.1 × 150 mm, 1.7 μm; Waters), and the mobile solutions consisted of water and acetonitrile with 9 mM ammonium formate at pH 8.0; the TripleTOF 5600 + was operated under positive ESI mode. The mass spectrometry analysis was changed between MS and data-dependent MS2 scans. After raw data pre-processing, peak finding/alignment, and peak annotation by MSDIAL software, metabolite identifications were supported by matching the retention time, accurate mass, and MS/MS fragmentation data to MSDIAL software database and online MS/MS libraries (Human Metabolome Database).

### Statistical analysis

#### Statistical analysis of clinical data

Data were analyzed using SPSS 16.0 and expressed as mean ± SD. Differences between 2 groups were analyzed using student’s t-test. The categorical data were analyzed by chi-square statistics. The significance level was set at *p* < 0.05.

#### Statistical analysis of multi-omics data

For each group pairing, the fold-change (FC) was calculated using the mean of each group and compared (e.g., mean of S-CAP vs mean of CON). A two-sided unpaired Welch’s t test was used to identify significant differences between groups. Statistically significant differentially abundant proteins (DAPs) and differentially abundant metabolites (DAMs) were identified using the following criteria: FC > 1.5 or FC < 0.67, and *p* < 0.05. *P*-values were adjusted for false discovery rate (FDR) using Benjamini and Hochberg. Partial least squares-discriminate analysis (PLS-DA) was conducted using MetaboAnalyst 4.0 and cross-validated using the tenfold unit variance scaling method.

Volcano plots were created based on FC and t tests, and the intensity data of these regions were used for GraphPad analysis and hierarchical clustering analysis. The cluster trend map is based on the Mfuzz R package [16], which can analyze the differential characteristics of proteins. The tool was able to identify potential patterns of change in the protein profile, and clustering proteins with similar patterns can help us understand the dynamic patterns of proteins. Bar plots for Gene Ontology (GO) enrichment were created in R 4.2.1. Heatmaps and signaling pathway analysis were performed using the Kyoto Encyclopedia of Genes and Genome (KEGG) database, Small Molecule Pathway Database (SMPDB) and Metaboanalyst 5.0. Mfuzz v.2.46.0. Connected networks were then visualized with String, a plug-in for Cytoscape (v.3.2.1).

### Selection of biomarker candidates

For biomarker selection and verification, a receiver operating characteristic (ROC) analysis was performed and the predictive power of each protein and metabolite was ranked according to the ROC area under curve (AUC) value. Next, 5 machine learning classifiers, including logistic regression, random forest, linear support vector machine, K-nearest neighbor, and decision tree were used to determine the best diagnostic model while the tenfold cross-validation method was used to evaluate their accuracy and error rate. Then, ROC curves were then applied to evaluate the accuracy of biomarker candidates in the validation set. Diagnostic parameters, including sensitivity and specificity, were also calculated.

## Results

### Sample cohort and experimental design

**Fig. 1:**
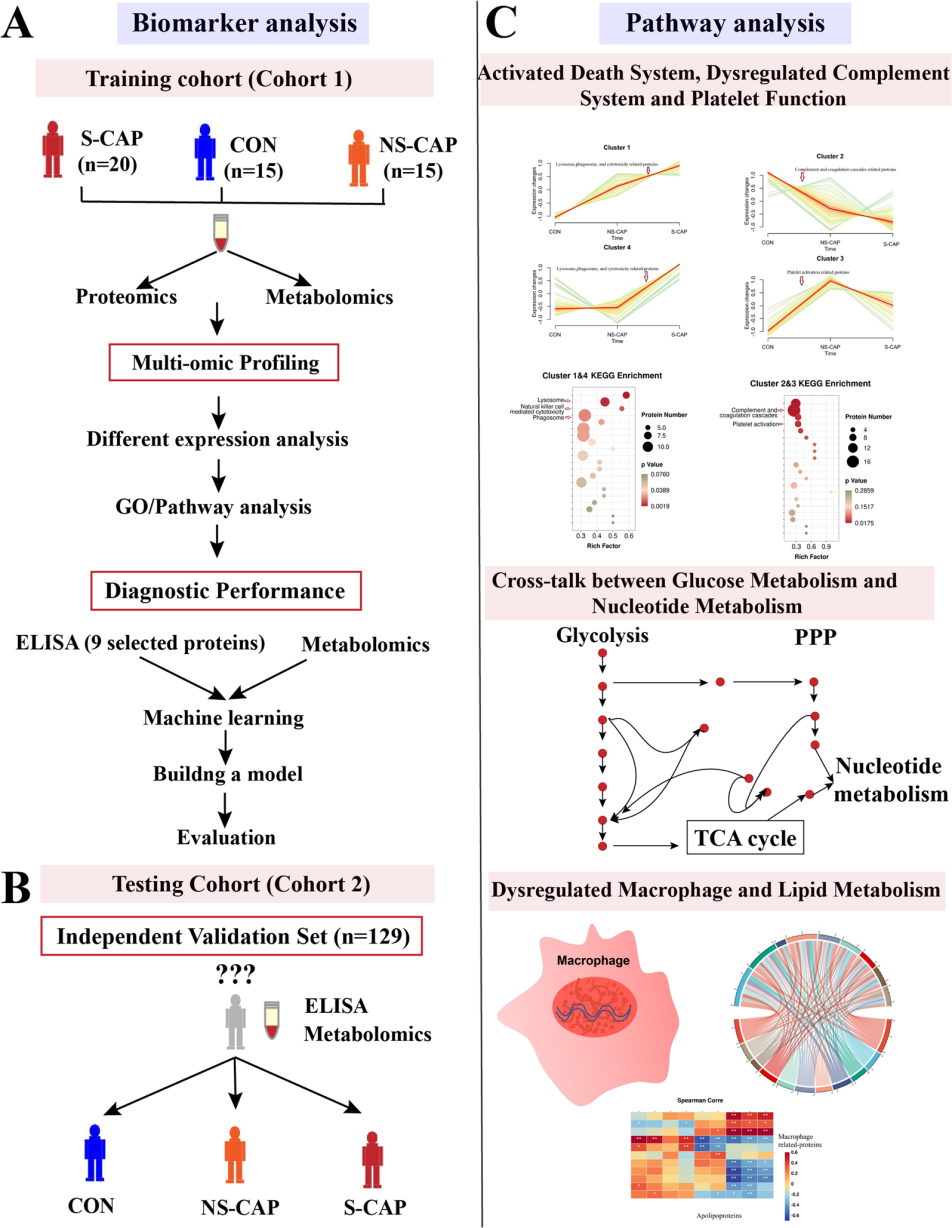
Study overview. **A** Study overview. 50 subjects including 20 S-CAPs, 15 NS-CAPs and 15 CONs from cohort 1 were recruited for proteomic and metabolomic analysis. Nine DAPs were verified with ELSIA in cohort 1. The DAPs and DAMs were then used to identify potential biomarker combinations for severe CAP diagnosis. **B** Selected biomarkers were verified using an independent cohort with 129 blinded subjects (cohort 2). **C** Protein-metabolite crosstalk was examined using integrated analysis. Proteomic and metabolomic signatures were analyzed to uncover the molecular profile for severe CAP

Proteomics and metabolomics were performed on serum samples taken from 50 participants, including 20 S-CAPs, 15 NS-CAPs and 15 CONs (Fig. 1A, cohort 1). Patients in the severe group had higher disease scores and clinical manifestations which required treatment in PICU. Based on the subjects from cohort 1, differentially abundant proteins (DAPs) and metabolites (DAMs) in CAP were identified using proteomics and metabolomics, with 9 DAPs verified using ELISA. A new serum biomarker panel was developed using machine learning algorithms to distinguish CAP from healthy controls as well as to identify severe cases of CAP. This panel included a combination of 2 proteins and 3 metabolites (Fig. 1A). Next, the serum biomarker panel was validated using ELISA and LC–MS/MS in an independent verification cohort (cohort 2) (Fig. 1B).

To determine the changes in host serum proteins, metabolites as well as the pathways which might contribute to the pathogenesis of severe CAP, cluster and pathway analyses were performed on the DAPs and DAMs identified (Fig. 1C). The relationship between DAPs and DAMs with clinical indices were also analyzed. The demographic characteristics and laboratory results of enrolled patients are shown in Additional file 2. These results were consistent with previous studies which showed several inflammation markers, such as Neu% and Lym% were associated with increased disease severity in CAP [11].

### Multi-omic profiling of CAP

#### Proteomic changes in CAPs

**Fig. 2:**
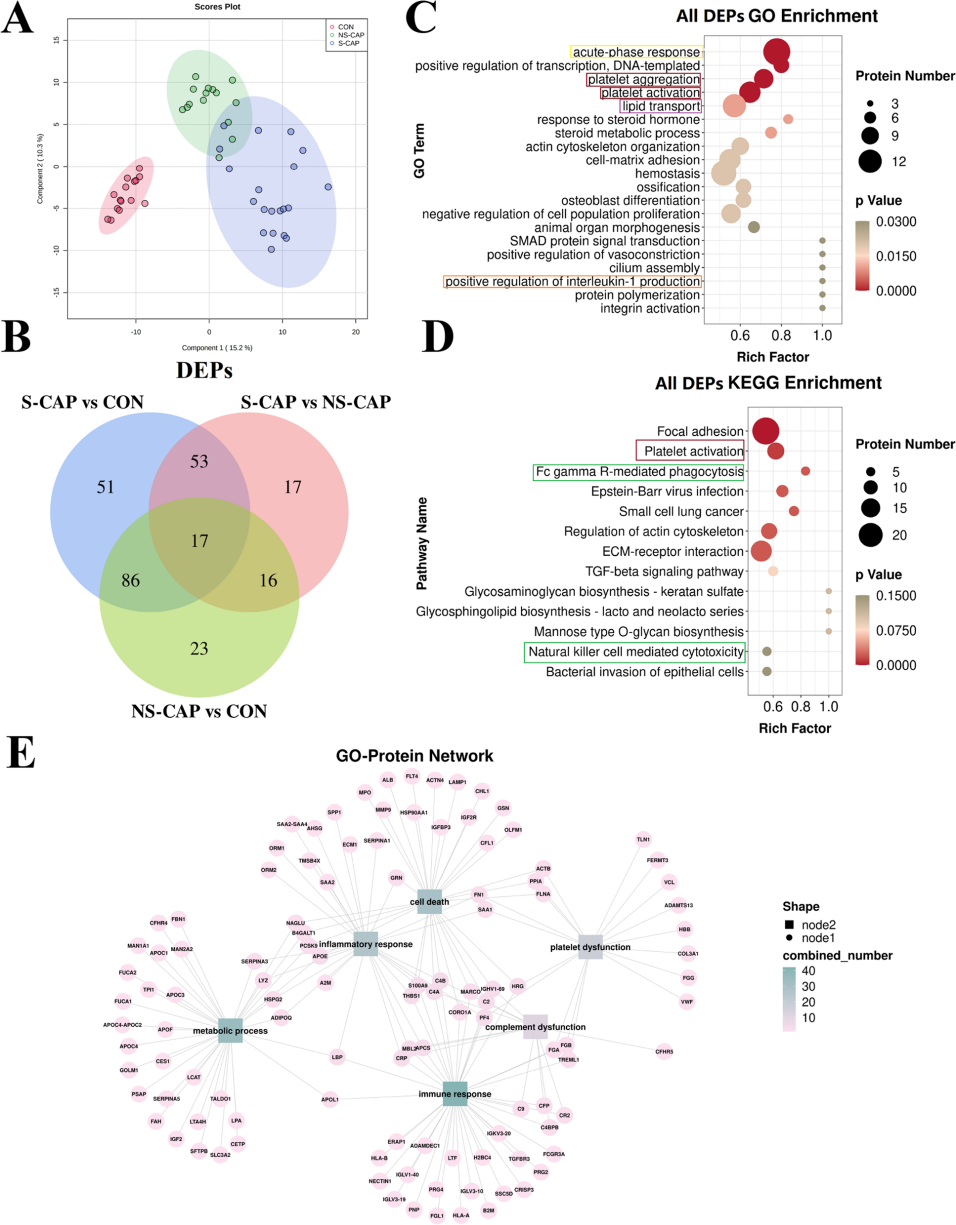
Identification of differentially abundant proteins in S-CAPs from cohort 1. **A** PLS-DA score plots for S-CAPs, NS-CAPs and CONs. **B** Venn diagram of the number of DAPs. **C** GO enrichment analysis for all DAPs with the top 20 GO terms shown. Yellow cycle highlights acute-phase response; Red cycle highlights platelet dysfunction; Orange cycle highlights immune response; Purple cycle highlights metabolic processes. **D** KEGG analysis for all DAPs with the top 13 KEGG terms shown. Red cycle highlights platelet dysfunction; Green cycle highlights cell death. **E** The interaction network for proteins involved in the cell death, inflammatory response, immune response, platelet dysfunction and metabolic pathways. Green squares represent pathways; purple circles represent the altered proteins; solid lines represent association between the pathways and proteins

Based on the LC–MS/MS data from cohort 1 samples, we identified a total of 7836 peptides (Additional file 3: Fig. S1A) and 514 proteins (Additional file 3: Fig. S1B). PLS-DA analysis (Fig. 2A) and volcano plots (Additional file 4: Fig. S2A–C) were used to visualize the DAPs. As shown in Fig. 2B, 263 proteins were differentially expressed among the three groups with 103 altered in the S-CAP group compared to the NS-CAP group (Additional file 5). This suggests that changes in serum proteins became more significant when disease was more severe. GO and KEGG pathway enrichment analyses were then performed on all DAPs. The GO terms (Fig. 2C) and KEGG pathways (Fig. 2D) were highly enriched for processes involved in inflammatory response (acute-phase response, yellow cycle), platelet dysfunction (red cycle), immune response (orange cycle), metabolic processes (lipid and carbohydrate metabolism, purple cycle), and cell death (green cycle). Furthermore, pairwise GO and KEGG (Additional file 4: Fig. S2D–I) analyses were also performed for DAPs between each group. Notably, the proteins belonging to these modules related to each other (Fig. 2E). Collectively, these results indicate that the altered serum proteins reflect the enhanced immune and inflammatory response, the dysregulation of platelets and metabolic processes, and cell death in S-CAPs.

#### Metabolomic alternations in CAPs

**Fig. 3:**
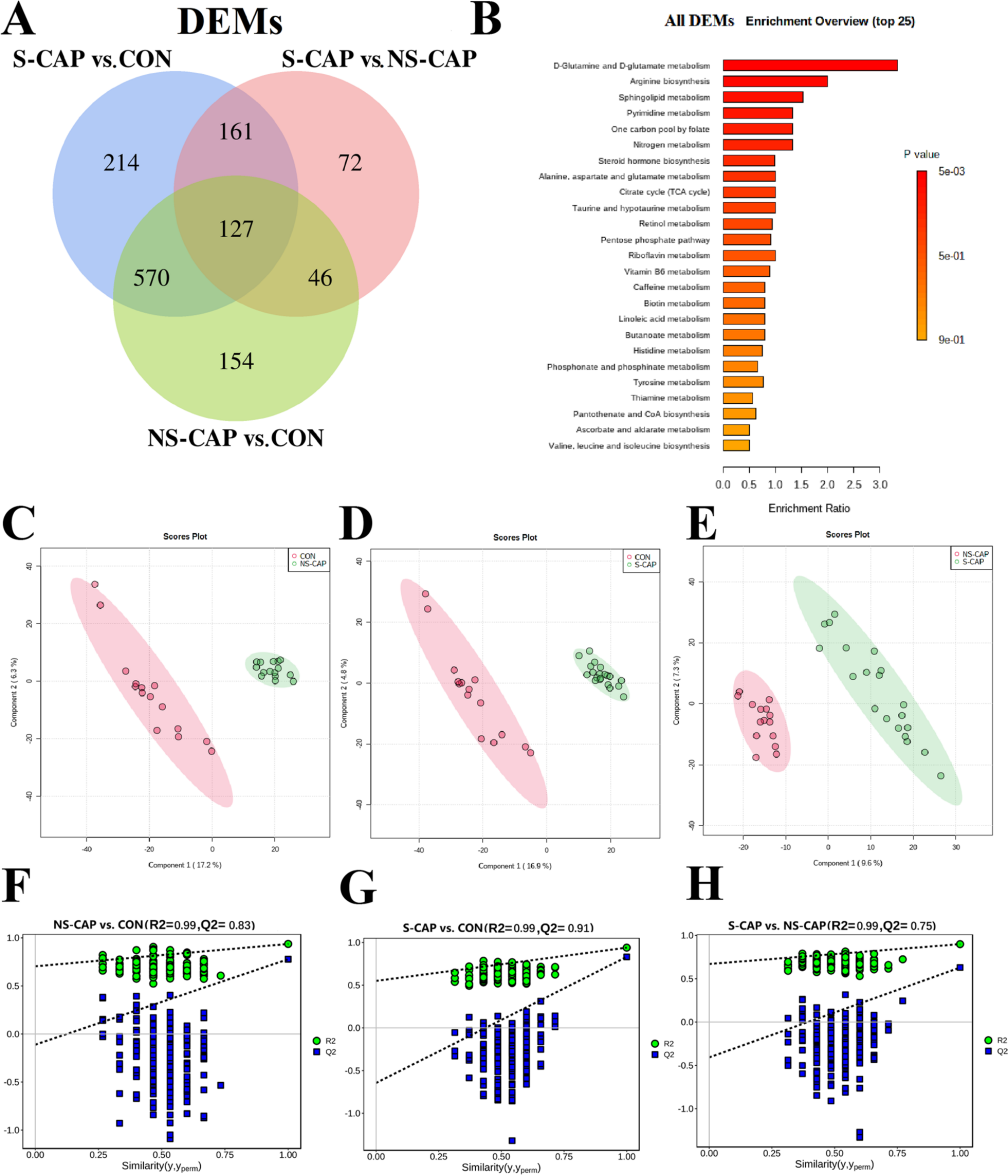
Identification of Differentially Abundant Metabolites in S-CAP from Cohort 1. **A** Venn diagram of the number of DAPs. **B** SMPDB analysis of the DAMs from cluster 1. Top 25 KEGG terms are shown. **C** PLS-DA score plots for NS-CAPs and CONs. (D) PLS-DA score plots for S-CAPs and CONs. **E** PLS-DA score plots for S-CAPs and NS-CAPs. **F** Parameters for assessing the quality of the PLS-DA model for NS-CAPs and CONs. **G** Parameters for assessing the quality of the PLS-DA model for S-CAPs and CONs. **H** Parameters for assessing the quality of the PLS-DA model for S-CAPs and CONs

For metabolomics, we identified a total of 38,841 peaks and 2687 metabolites from cohort 1 including amino acids, lipids and other important serum metabolites. Of these, 1344 DAMs were significantly altered among the 3 groups and 127 were overlapping (Fig. 3A and Additional file 5). SMPDB analysis indicated a significant impact of CAP on D-glutamine and D-glutamate metabolism and arginine biosynthesis (Fig. 3B). PLS-DA models were used to visualize the separation of NS-CAPs with CONs (Fig. 3C), S-CAPs with CONs (Fig. 3D), and S-CAPs with NS-CAPs (Fig. 3E). Clear differences were observed for each group, with cumulative R2 = 0.99 and Q2 = 0.83 between the NS-CAP and CON groups (Fig. 3F), with cumulative R2 = 0.99 and Q2 = 0.91 between the S-CAP and CON groups (Fig. 3G), and with cumulative R2 = 0.99 and Q2 = 0.75 between the S-CAP and NS-CAP groups (Fig. 3H). The separation of the 3 groups based on DAMs suggest metabolic dysregulation is involved in the pathogenesis of CAPs which is augmented with severe disease.

### Identification of a serum biomarker panel for severe CAP

Based on the serum proteomic data from cohort 1, we selected nine potential candidate biomarkers (ADIPOQ, ALB, AGT, PROC, LBP, HRG, FN1, CRP and APOC1) for verification with ELISA. The criteria for selection were as follows: (1) high FC; (2) high ROC value; and (3) associated with immunity, infection or death-related process. As expected, significant differences were observed with ratios consistent with the proteomic data (Additional file 6: Fig. S3).

**Fig. 4:**
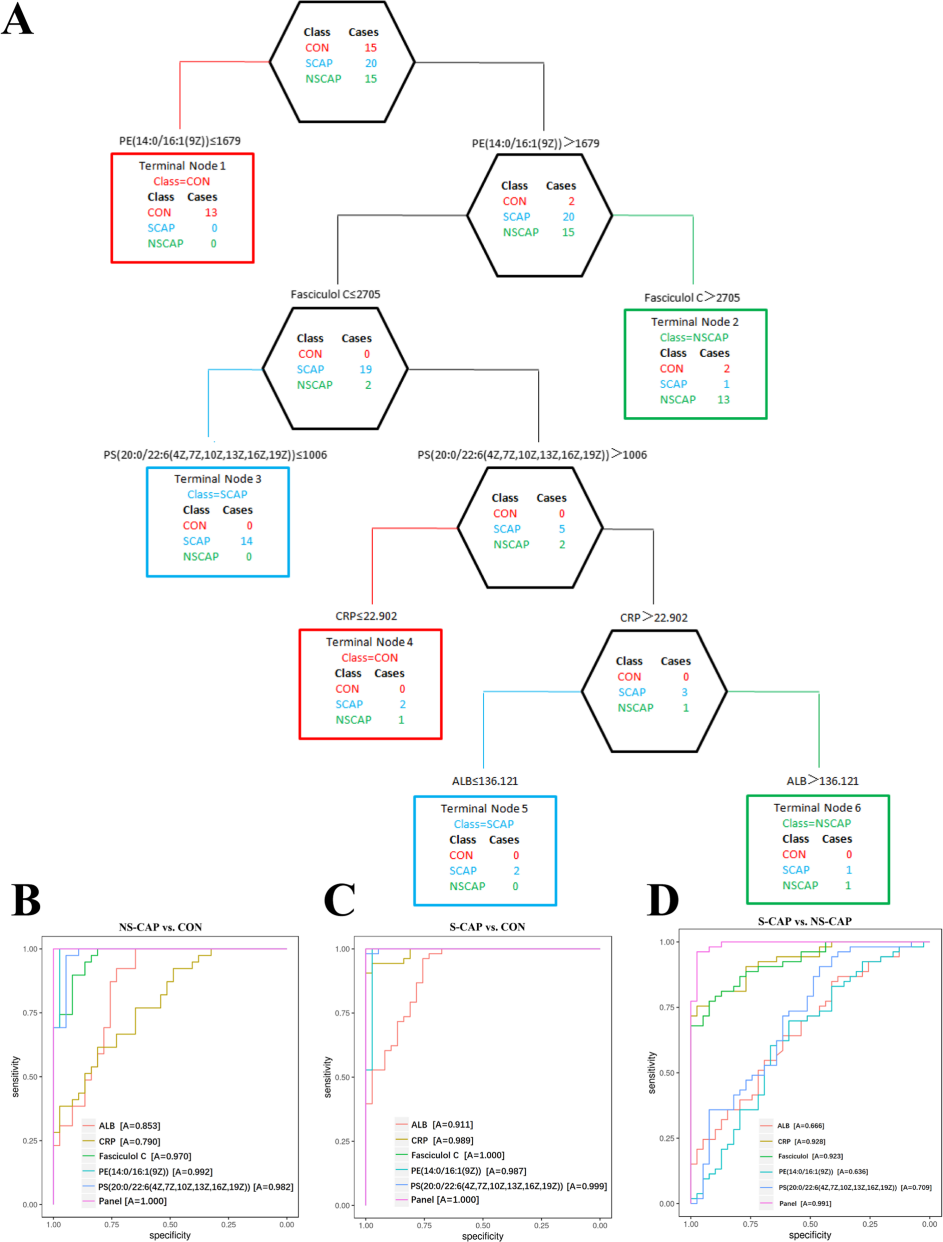
Identification and verification of potential biomarkers for classification of S-CAPs. **A** Classification and regression tree analysis using 2 DAPs and 3 DAMs with 6 terminal nodes. The selected splitting variables are shown in the nodes. **B** AUC values for 5 biomarkers and the combined panel were calculated to differentiate NS-CAPs from CONs in cohort 2. **C** AUC values for 5 biomarkers and the combined panel were calculated to differentiate S-CAPs from CONs in cohort 2. **D** AUC values for 5 biomarkers and the combined panel were calculated to differentiate S-CAPs from NS-CAPs in cohort 2

Next, based on the ELISA and metabolomics data, we developed a new computational pipeline to identify potential biomarker combinations for diagnosis of S-CAPs cases. For the pipeline, nine verified DAPs were used to build a protein classification tree and two DAPs were eventually selected as the best combination (Additional file 7: Fig. S4A). Similarly, 4 DAMs with AUC > 0.9 were used to build a metabolite classification tree and 3 DAMs were selected (Additional file 7: Fig. S4B). Next, we combined the selected DAPs and DAMs for best panel selection. As presented in Fig. 4A, the optimal marker set, included 2 proteins (CRP, LBP) and 3 metabolites [Fasciculol C, PE (14:0/16:1(19Z)), PS (20:0/22:6(4Z, 7Z, 10Z, 13Z, 16Z, 19Z))] with higher significance than other panels (Additional file 7: Figs. S4A and B). This model was able to completely distinguish S-CAPs and NS-CAPs from CONs, with 100% sensitivity and 100% specificity. Moreover, it was also able to discriminate S-CAPs from NS-CAPs, which suggests that this marker set has the potential to differentiate severe pneumonia in children.

### Independent validation of serum biomarkers

To test the accuracy of the selected biomarker panel for S-CAP diagnosis, we used an independent cohort of 129 subjects, including 53 S-CAPs, 39 NS-CAPs, and 37 CONs. Serum samples were subjected to ELISA (Additional file 6: Fig. S3) and LC–MS/MS to detect levels of proteins and metabolites, respectively. The AUC value of this panel to distinguish NS-CAPs (Fig. 4B) and S-CAPs (Fig. 4C) from CONs was 100%. Moreover, the AUC value of this panel to distinguish S-CAP from NS-CAPs was 0.991 (Fig. 4D). When each protein/metabolite was compared individually as well as in combination (Additional file 7: Fig. S4C–E), the AUC values showed that even when used alone, the DAPs and DAMs were still informative to distinguish between different groups in most cases (Fig. 4B–D). Thus, our results confirmed the accuracy of the proteomic and metabolomics data in cohort 1 and, more importantly, validated the serum biomarker panel identified in this study as having promising potential to clinically identify S-CAP in children.

### Activated death system, dysregulated complement system and platelet function in S-CAP cases

**Fig. 5:**
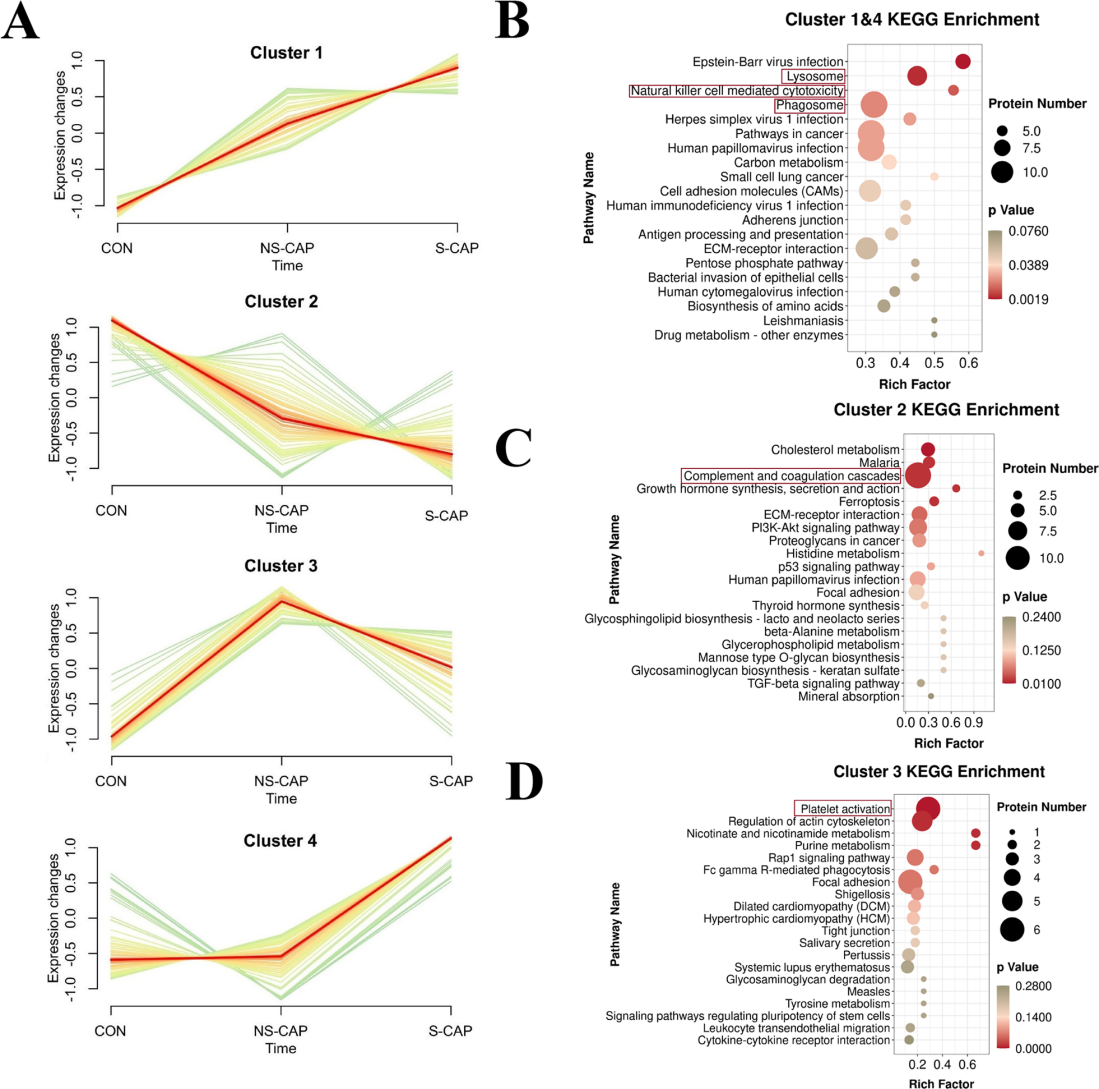
Activated death system, dysregulated complement system and platelet function in S-CAP cases. **A** Hierarchical clustering illustrating four DAP patterns across three groups. The red line is the center line of the trend for each gene cluster. **B** KEGG terms enriched in cluster 1 and cluster 4. Top 20 KEGG terms are shown. Red lines highlight cell death-related pathways. **C** KEGG terms enriched in cluster 2. Top 20 KEGG terms are shown. Red lines highlight complement and coagulation cascade pathways. **D** KEGG terms enriched in cluster 3. Top 20 KEGG terms are shown. Red lines highlight platelet activation pathway

From the DAPs identified (Fig. 2B), three expression patterns including two increasing clusters (cluster 1 and cluster 4), a decreasing cluster (cluster 2) and an inverted “V” cluster (cluster 3) were observed across the different groups (Fig. 5A and Additional file 8).

We then performed KEGG pathway enrichment analyses on the DAPs from each cluster pattern. Interestingly, DAPs in the increasing clusters (cluster 1 and cluster 4) were enriched in proteins associated with the lysosome, natural killer cell mediated cytotoxicity and phagosome pathways, suggesting that these death processes contributed to the development of severity (Fig. 5B and Additional file 8). Higher expression of death-related proteins suggests these processes may be involved in the development of severe pneumonia (Additional file 9: Fig. S5A). Many cell death-related proteins formed a correlated network with DAPs in other cell death-related pathways (Additional file 9: Fig. S5B) and were positively correlated with disease severity (Additional file 9: Fig. S5C).

For the decreasing cluster (cluster 2), DAPs were enriched in cholesterol metabolism, malaria, and complement and coagulation cascades (Fig. 5C and Additional file 8). Most complement-related proteins [complement factor H-related protein (CFHR)3, CFHR4, CFHR5] and coagulation-related proteins [alpha-2-macroglobulin (A2M), PROC, heparin cofactor 2 (SERPIND1)] were decreased in CAPs, with some lower in S-CAPs compared to NS-CAPs (Additional file 9: Fig. S5D). This disordered complement and coagulation cascade response might be associated with the occurrence of multi-organ dysfunction syndrome which is frequently fatal in severe patients.

For the inverted “V” cluster (cluster 3), these contain proteins primarily involved in platelet activation (Fig. 5D and Additional file 8). In this study, proteins involved in platelet activation were increased in mild CAPs but significantly decreased in severe cases. The expressions of platelet-related proteins were negatively associated with disease severity (Additional file 9: Fig. S5E). Additionally, levels of fibrinogen alpha (FGA) and fibrinogen beta (FGB) were positively correlated with FDP levels (clinical index, Additional file 9: Fig. S5F). Collectively, these DAPs in S-CAPs indicate suppression of the complement system and platelet function in severe disease, which suggests that tissue remodeling might be severely inhibited during this period.

### Cross-talk between glucose metabolism and nucleotide metabolism implicated in progression to severe disease in CAP

To gain an insight into the pathogenesis of S-CAP, we used metabolomics data (combined from cohort 1 and 2) to further investigate changes associated with dysregulated function and severe disease (Additional file 10: Fig. S6).

**Fig. 6:**
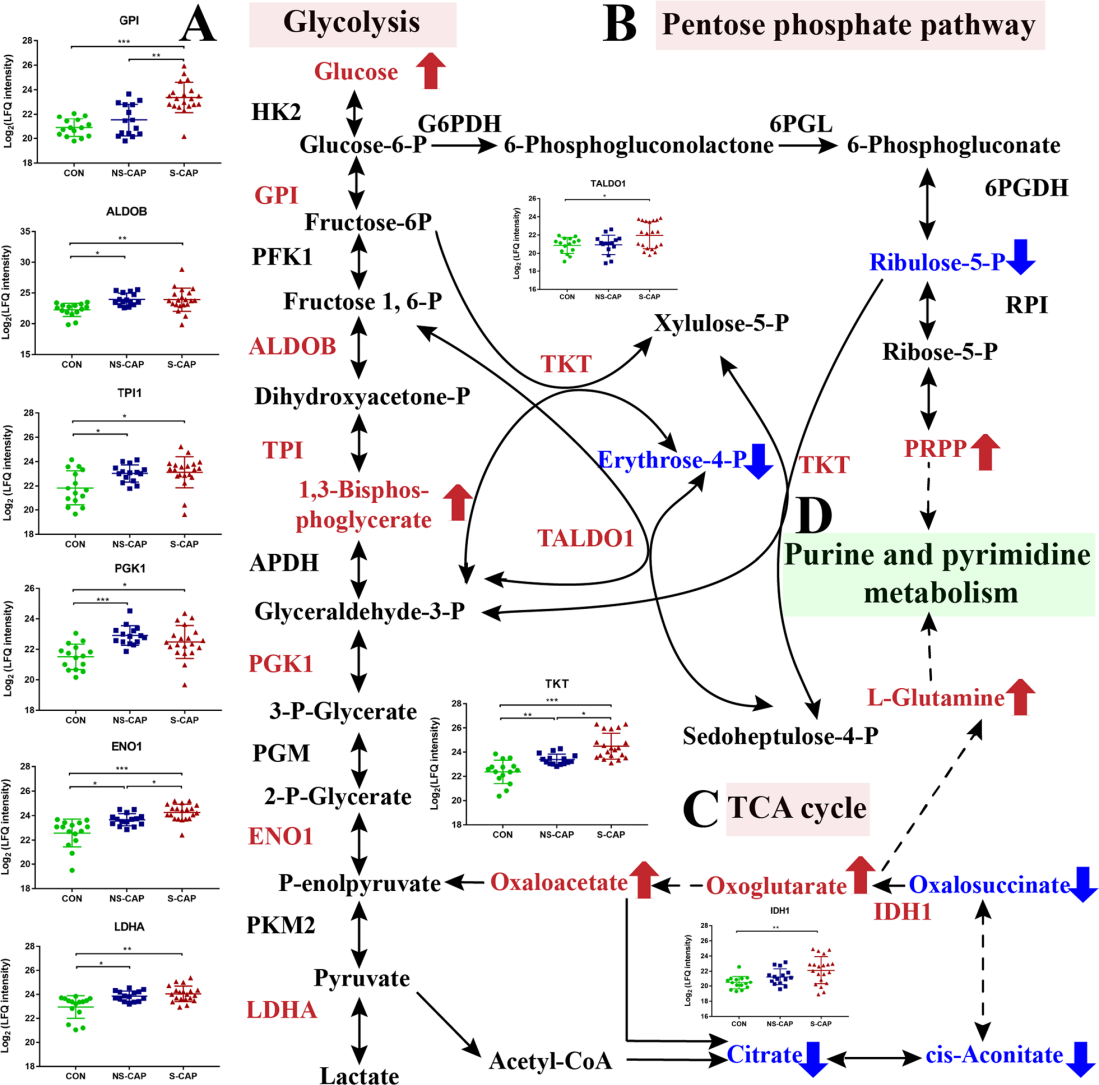
Cross-talk between glucose metabolism and nucleotide metabolism implicated in progression to severe disease in CAP. **A** Glycolysis and **B** Pentose phosphate pathway (PPP) were activated during the initial onset of CAP and progression toward severe disease. Most enzymes involved in glycolysis were significantly upregulated in CAPs. **C** Circulating levels of TCA metabolites in serum. Increased proteins and metabolites are labeled in red. Decreased proteins and metabolites were labeled in blue. Statistical significance was determined using the FDR-adjusted *p*-value. *p < 0.05; **p < 0.01; ***p < 0.00

Glucose is the primary energy source for immune cells and a key player for pathogen proliferation and inflammation. In this study, most enzymes involved in glycolysis, including glucose-6-phosphate isomerase (GPI), fructose-bisphosphate aldolase (ALDOB), triosephosphate isomerase (TPI1), phosphoglycerate kinase 1 (PGK1), alpha-enolase (ENO1) and l-lactate dehydrogenase A (LDHA) were significantly upregulated in CAPs (Fig. 6A). Compared to NS-CAP cases, some of these enzymes were further elevated in S-CAP. Consistent with the proteomics results, metabolomics also found significant increase in glycolysis intermediary metabolites including glucose and 1,3-bisphosphoglycerate in S-CAP (Fig. 6A). Activation of the glycolysis pathway, which is necessary for virus replication, were also observed in severe COVID-19 patients [27].

Cross-talk between glucose metabolism and nucleotide metabolism is essential as both the PPP and TCA cycle contribute to nucleotide formation by increasing the supply of glutamate and/or phosphoribosyl pyrophosphate (PRPP) [28, 29]. Here, the levels of transaldolase (TALDO1) and transketolase (TKT), which acts as a bridge between glycolysis and the TCA cycle [28], were up-regulated in CAPs, especially in S-CAPs (Fig. 6B). Moreover, the level of PRPP, which is a source of PPP for purine and pyrimidine metabolism [30], was almost 70 times and 66.7 times higher in the S-CAPs and NS-CAPs group compared CONs (Fig. 6B). A significant upregulation of glutamine and TCA intermediary metabolites (oxaloacetate and oxoglutarate) as well as downregulation of citrate, cis-Aconitate and oxalosuccinate were also observed in CAPs. Levels of isocitrate dehydrogenase 1 (IDH1) were also significantly increased in NS-CAPs and S-CAPs (Fig. 6C). In addition, we analyzed the amount of nucleotide intermediates in CAPs and found that many purine metabolic intermediates (dAMP, dGMP, guanosine, deoxyinosine and inosine, Additional file 11: Fig. S7) and pyrimidine metabolic intermediates (CMP, dCMP, dUMP, dUTP and dTDP, Additional file 12: Fig. S8) were also significantly increased. Collectively, this cross-talk between glucose and nucleotide metabolism may provide metabolic intermediates and energy for inflammation.

#### Dysregulated macrophage and lipid metabolism in CAP

**Fig. 7:**
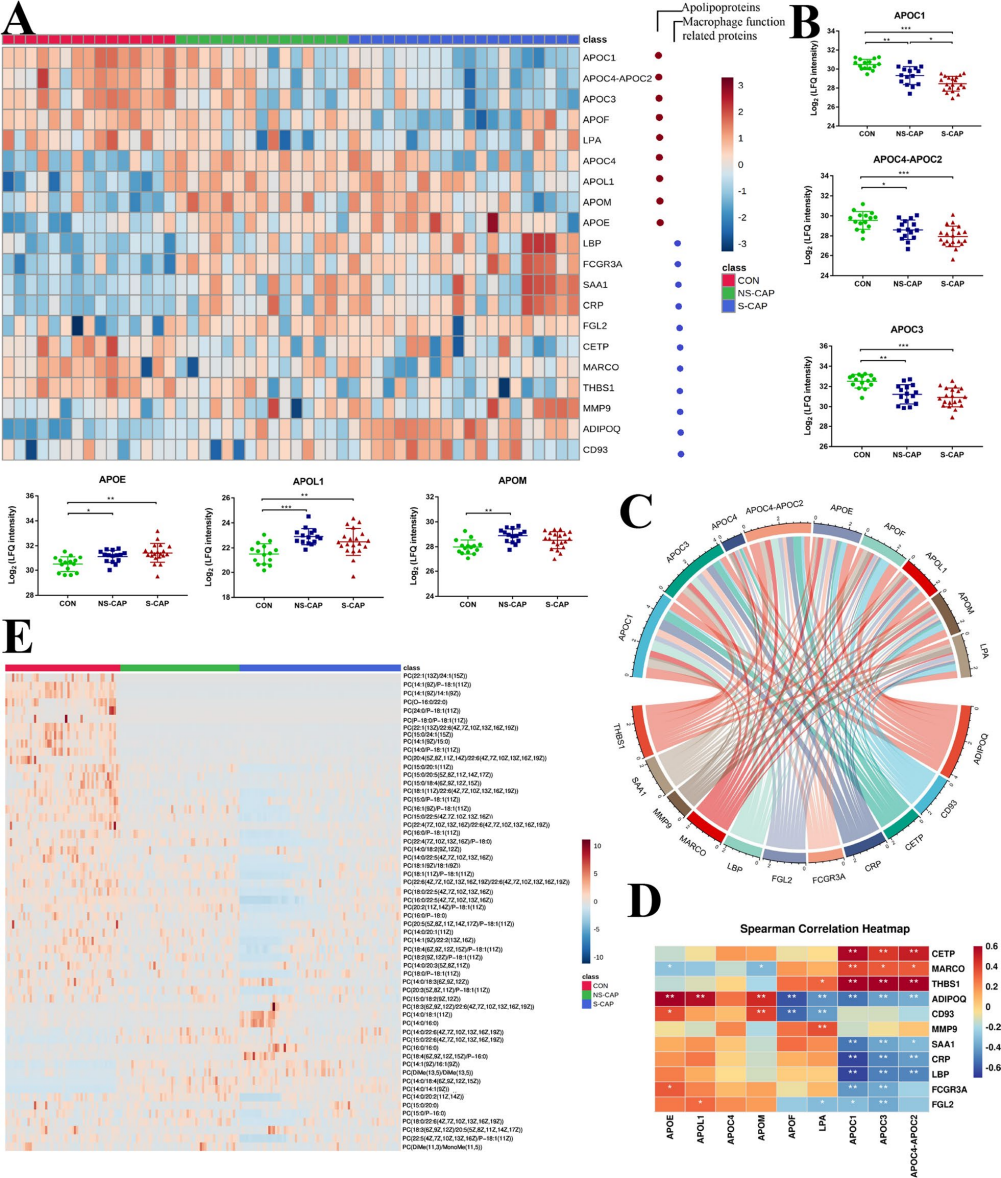
Dysregulated lipid metabolism in CAP. **A** Heatmap showing expression levels of apolipoproteins in CONs, S-CAPs, and NS-CAPs. **B** Representative apolipoprotein expression changes across 3 groups. Square and bars represent the mean and standard deviation, respectively. Statistical significance was determined using the FDR-adjusted *p*-value. *p < 0.05; **p < 0.01; ***p < 0.001. **C** The interaction network for apolipoproteins and proteins associated with macrophage function. **D** Correlation analysis of inflammatory-associated proteins and apolipoproteins. Red and blue numbers represent positive and negative correlation, respectively. * means correlation *p* value < 0.05. ** means correlation *p* value < 0.01. **E** Heatmap of DAMs that are associated with fatty acyls, glycerolipids, glycerophospholipids, prenol lipids, sphingolipids, steroid and steroid derivatives

In addition to glucose and nucleotide metabolism, proteins involved in lipid metabolism were also altered in CAPs. Expression of multiple apolipoproteins including APOC1, APOC4-APOC2, APOC3, APOC4, APOF, APOL1, APOM and APOE were changed (Fig. 7A). Most of these apolipoproteins were down-regulated and associated with macrophage function. Decreased expression of APOC (APOC1, APOC4-APOC2 and APOC3) in CAP patients may contribute to macrophage modulation (Fig. 7B). The expression of APOE, APOL1 and APOM was increased in both NS-CAPs and S-CAPs (Fig. 7B). Dysregulation of serum APOL1 and APOM has also been reported in COVID-19 patients [7]. Moreover, macrophage-related proteins [such as Macrophage receptor (MARCO)] were also differentially expressed and these proteins were correlated with apolipoproteins in terms of function and expression (Fig. 7C, D).

Macrophages are closely associated with lipid metabolism. Macrophages are known to regulate lipid synthesis after exposure to inflammatory stimuli, which amplify the inflammatory response [33]. It has been reported that macrophage exposed to microbial stimuli upregulate the synthesis of phosphatidylcholine (PC) [33]. However, in this study, we found many PCs were downregulated in CAPs, especially for S-CAPs (Fig. 7E). This suggests that macrophage function might be impaired in children with severe pneumonia.

## Discussion

Severe pediatric CAP is a critical public health threat to children’s health. Although bacterial and viral infections may lead to different results, both present with symptoms of pneumonia. Healthcare associated pneumonia is no longer recognized as a distinct entity, but as a form of CAP, and there is increasing evidence of bacterial and virus as etiological agents of CAP. Due to the complexity and heterogeneity of the disease, diagnosis of CAP, especially for severe CAP, remains a clinical challenge. Therefore, it is important to identify early biomarkers that can detect the severity of CAP. For this purpose, we applied proteomics and metabolomics to test the serum protein and metabolite changes associated with severe CAP. To our knowledge, this is the first study to combine proteomic and metabolomic data obtained from children with CAP and different disease severity. Our study identified 2 proteins (CRP, LBP) and 3 metabolites [Fasciculol C, PE (14:0/16:1(19Z)), PS (20:0/22:6(4Z, 7Z, 10Z, 13Z, 16Z, 19Z))], which are good candidates to identify severe CAP cases from non-severe CAP cases and controls. These candidates were further validated in an independent cohort.

In this study, the proteomics and metabolomics data generated also enabled a systematic analysis of the molecular pathology in CAP. The development of children's lung function is not perfect, so age is likely to be an important factor affecting metabolism and morbidity. Therefore, we age-matched the cases and controls to minimize the influence of age on protein and metabolite abundance in each group. Significantly DAPs were identified to be involved in essential biological processes such as cell death, the complement system, coagulation cascades, platelet function and metabolic dysregulation. Our results are consistent with previous findings that severe CAP cases are frequently associated with acute respiratory distress syndrome, sepsis, and multi-organ injury [34], which were pathophysiologically associated with cell death activation pathway, intravascular coagulation and microthrombosis [34]. Our data revealed the molecular changes in CAP sera, which could potentially reflect the occurrence of cell damage in CAP. Here, we observed that severe CAP patients are often accompanied by tissue damage and inflammation. Higher expression of lysosome-related proteins, cytotoxicity-related proteins and phagosome-related proteins were observed in S-CAPs, suggesting that various cell death pathways contribute to the development of severe pneumonia. Lysosomes which are found in pre-existing endolysosomes or autolysosomes act as an important bridge between autophagy and endocytosis [35]. Thus, as an important regulator of cell death, lysosomes, cytotoxicity proteins and the phagosome may be involved in exacerbating CAP leading to the development of severe disease.

Our data also observed activation of the complement system and inflammation system in CAPs. Here, multiple acute phase proteins such as CRP and complement-related proteins were upregulated in CAPs. It has been reported that CRP assists in activation of the complement system [36]. This induces the production of cytokines and chemokines, potentially resulting in a ‘‘cytokine storm’’ [36]; and also recruits macrophages from the peripheral blood, which may lead to acute lung injury. Since ~ 50% of platelets are produced in the lungs [37], these platelets may help to aggravate lung injury and further induce cytokine storm. For example, C4BPB [18] and F11 [19] which are regulators of complement system were significantly decreased in S-CAP cases. PROC, which interacts with C4BP [20], was also downregulated in S-CAPs. Moreover, CFHR3 [21], CFHR4, CFHR5 [22] and CR2 were also decreased in S-CAP patients compared to CONs. Complement and coagulation, together with platelet dysfunction, act as the linchpin in events leading to thromboinflammation [17]. Declining platelet count has also been associated with poor outcomes in CAP patients [23]. Two of the most intriguing proteins downregulated in severe patients were vasodilator-stimulated phosphoprotein (VASP) and integrin alpha-IIb (ITGA2B). VASP is an actin regulatory protein implicated in platelet adhesion [24] while ITGA2B encodes aIIb and is an important gene associated with COVID-19-related stroke [25]. In addition, the expression of most complement proteins, coagulation cascade proteins and platelet-related proteins were negatively associated with disease severity. Interestingly, the levels of platelet-related proteins, such as collagen alpha-1(I) (COL1A1), ITGA2B, fermitin family homolog 3 (FERMT3), talin-1 (TLN1) and VASP were positively correlated with TT levels, while negatively correlated with FIB levels, which are essential clinical indexes. Additionally, levels of fibrinogen alpha (FGA) and fibrinogen beta (FGB) were positively correlated with FDP levels (clinical index). Recently, increasing evidence indicates a potential cross-talk between complement factors and platelet activation, contributing to the pathophysiology of diseases and subsequent tissue remodeling processes [17]. Therefore, activation of the cell death pathway, the inflammatory response and a dysregulated complement, coagulation cascade and platelet function are predicted to cause tissue damage in children with CAP.

Cross-talk between glucose metabolism and nucleotide metabolism were observed in CAP cases. Nucleotides are the building blocks for DNA and RNA synthesis. Glucose metabolic pathways such as the PPP and TCA cycle promote nucleotide formation by increasing the supply of glutamate and/or PRPP [29, 38]. In this study, the levels of PRPP and glutamine were significantly upregulated in NS-CAPs and S-CAPs. Moreover, the nucleotide (CMP) and most deoxynucleotide (dAMP, dGMP, dCMP, dUMP) were also elevated in NS-CAP and/or S-CAPs. One explanation for this “cross-talk” might reflect increased DNA and RNA synthesis in CAP patients due to proliferation of immune cells as nucleotides are required for replication [29]. Modulating nucleotide metabolism may also increase the host immune response against pathogen attack [29, 39]. Furthermore, increased nucleotides and deoxynucleotides in the serum suggests higher RNA turnover and DNA degradation possibly due to apoptosis of host cells or immune cells. Consistent with previous reports, RNA turnover and DNA degradation are increased in inflammatory diseases [40, 41]. The role of increased (deoxy)nucleotides in the pathogenesis of pneumonia requires further research; however, it is possible that higher levels of nucleotides lead to unbalanced deoxyribonucleotide pools which, in turn, contribute to the progression to severe CAP.

In addition to our findings of altered glucose and nucleotide metabolism in CAPs, we also uncovered dysregulated metabolites for lipid metabolism which are important for regulation of signal transduction and immune activation processes. Previously, Ning et al. [11] suggested that sphingolipid metabolism was significantly affected in CAPs, and that lipid dysfunction was one of the potential pathological mechanisms. In another study on serum metabolites and lipid alterations in CAPs, sphingolipids were strongly correlated with respiratory function, the cardiovascular system and liver function [42]. Similarly, our data also showed that lower sphingolipids were detected in both NS and S-CAP patients. In addition, dysregulated expression of APOM was reported to be associated with virus infection [7]. This finding was consistent with our finding that the levels of apolipoproteins, which are involved in the transport and redistribution of lipids, were significantly dysregulated in both NS-CAP and S-CAP patients. Moreover, it is known that pulmonary surfactant is a protein-lipid mixture secreted by type-II alveolar epithelial cells. Impaired surfactant function in lung is thought to be an essential mechanism for pneumonia after pathogen infection. Thus, altered lipid metabolism in this study might have also been induced by surfactant metabolism dysfunction after pathogen infection. Furthermore, it has been reported that CAPs with pulmonary diffusing capacity affect oxygen transport and mitochondria changes in the β-oxidation pathway in children, especially young children. The previous study also reported that lipid catabolism can been improved by enhanced lipolytic and fatty acid β-oxidation pathways [43]. Thus, we hypothesize that lipid metabolism and anaerobic pathways can be altered by the damage of pulmonary diffusing capacity due to lack of adequate oxygen, as well as beta-oxidation pathways in mitochondria due to CAP. Together, these data collectively indicate that dysregulated lipid metabolism is involved in the pathological mechanism of CAP disease progression.

There are still some limitations to this study which needs to be considered. Although our samples were age-matched, there may still be other genetic, clinical or environmental confounding factors such as pathogen type that may not have been detected or controlled for. Furthermore, although our results were verified using an independent cohort, further verification using larger samples sizes are still needed.

In conclusion, this study provides a systematic proteomic and metabolomic investigation of serum samples taken from severe and mild CAP patients as well as control groups. We demonstrated the potential of a panel of serum proteins and metabolites that can identify CAP cases which may progress into severe pneumonia. Although we successfully validated our serum biomarker panel in an independent testing cohort, the two cohort sizes are small and may require larger samples sizes to confirm our findings. Our data also laid out the molecular profile of serum changes in pediatric CAP, which may provide additional useful diagnostic markers and information for the development of therapeutic interventions in children who develop severe pneumonia.

## Supplementary Information

**Additional file 1**. **Table S1**. Additional characteristics and pathogenic types of S-CAPs, NS-CAPs and CONs.

**Additional file 2**. **Table S2**. The clinical information and conducted biochemical laboratory tests.

**Additional file 3**. **Fig. S1**. Quality control and differentially abundant proteins (DAPs) in different samples. Distribution of the number of **A** quantified peptides and **B** proteins in the 50 serum samples from cohort 1.

**Additional file 4**. **Fig. S2**. DAPs in different pairwise comparison. Volcano plot comparing protein expression in **A** NS-CAP vs. CON, **B** S-CAP vs. CON and **C** S-CAP vs. NS-CAP. Proteins with FC >1.5 or <0.67 with *P* value <0.05 were considered to be significant DAPs. Number of significantly down- (green) and up- (red) regulated proteins are shown on top. GO-BP analysis of the DAPs from **D** NS-CAP vs. CON, **F** S-CAP vs. CON and **H** S-CAP vs. NS-CAP. KEGG analysis of the DAPs from **E** NS-CAP vs. CON, **G** S-CAP vs. CON and **I** S-CAP vs. NS-CAP. Top 20 terms are shown with red lines highlighting platelet-related pathways, yellow lines highlighting inflammatory-related pathways, green lines highlighting cell death-related pathways and purple lines highlighting metabolism-related pathways.

**Additional file 5**. **Table S3**. Differentially abundant proteins (DAPs) and Different abundant metabolites (DAMs) of cohort 1

**Additional file 6**. **Fig. S3**. Selected DAPs Verified using ELISA in Samples from Cohort 1 and Cohort 2. Protein levels for 9 selected DAPs were verified using ELISA. Statistical significance was determined by Student’s t test. *p < 0.05; **p < 0.01; ***p < 0.001.

**Additional file 7. Fig. S4:** Identification and verification of potential biomarker panels for classification of severe CAPs. **A** Classification and regression tree analysis using 2 DAPs with 2 terminal nodes. **B** Classification and regression tree analysis using 3 DAMs with 3 terminal nodes. **C** AUC values for the 2 combined panels were calculated to differentiate NS-CAPs from CONs in cohort 2. **D** AUC values for the 2 combined panels were calculated to differentiate S-CAPs from CONs in cohort 2. **D** AUC values for the 2 combined panels were calculated to differentiate S-CAPs from NS-CAPs in cohort 2.

**Additional file 8**. **Table S4**. Clusters of Differentially abundant proteins (DAPs) and KEGG analysis of DAP clusters

**Additional file 9**. **Fig. S5**. Details of activated death system, dysregulated complement system and platelet function in S-CAP cases. **A** Heatmap showing expression levels of DAPs related to lysosome, nature killer cell mediated cytotoxicity, and phagosome. **B** The interaction network for proteins involved in the lysosome, nature killer cell mediated cytotoxicity, and phagosome pathways. **C** Correlation of death-related DAPs and pediatric critical illness score (PCIS). *x* axis depicts Spearman’s correlation coefficients. **D** Heatmap showing expression levels of proteins related to complement, coagulation cascades and platelet activation. **E** DAPs associated with the platelet activation pathway were correlated to PCIS. *x* axis shows Spearman’s correlation coefficients. **F** Spearman correlation heatmap between expression levels of DAPs associated with platelet-related pathways and clinical indices associated with platelet function. * means correlation *p* value < 0.05. ** means correlation *p* value < 0.01. Red means positive correlation. Blue means negative correlation.

**Additional file 10**. **Fig. S6**. Study overview of differentially abundant metabolites (DAMs) in different groups. Metabolomics data from cohort 1 and cohort 2 combined and analyzed.

**Additional file 11**. **Fig. S7**. Activation of Purine Metabolism in CAPs. Many purine metabolic intermediates (dAMP, dGMP, guanosine, deoxyinosine, and inosine) were significantly increased (as shown in red). Decreased proteins and metabolites were labeled in blue. Statistical significance was determined using the FDR-adjusted *p*-values. *p < 0.05; **p < 0.01; ***p < 0.001.

**Additional file 12**. **Fig. S8**. Activation of pyrimidine metabolism in CAPs. Many pyrimidine metabolic intermediates (CMP, dCMP, dUMP, dUTP and dTDP were significantly increased (as shown in red). Decreased proteins and metabolites were labeled in blue. Statistical significance was determined using the FDR-adjusted *p*-values. *p < 0.05; **p < 0.01; ***p < 0.001.

## Data Availability

Correspondence and requests for data and materials should be addressed to Prof. Yi Wang and Prof. Jieqiong Li.
