# Supplementary material for: Serum-integrated omics reveal the host response landscape for severe pediatric community-acquired pneumonia
Source: Crit Care. 2023 Mar 1;27:79. doi: 10.1186/s13054-023-04378-w (PMC9976684; doi:10.1186/s13054-023-04378-w)
Supplement: Supplementary file 2 — Additional file 2. Table S2. The clinical information and conducted biochemical laboratory tests. [file 13054_2023_4378_MOESM2_ESM.docx]

Supplementary Data 2: The clinical information and conducted biochemical laboratory tests.

| Characteristics | Cohort 1 (n=50) | | | | Cohort 2 (n=129) | | | |
| --- | --- | --- | --- | --- | --- | --- | --- | --- |
|  | S-CAP | NS-CAP | CON | P value ^a^ | S-CAP | NS-CAP | CON | P value ^a^ |
| Number | 20 | 15 | 15 | / | 53 | 39 | 37 | / |
| Gender (male/female) | 12/8 | 9/6 | 8/7 | 0.705 | 37/16 | 27/12 | 25/12 | 0.797 |
| Age (years) ^b^ | 2.0±3.4 | 2.0±1.2 | 2.9±3.0 | 0.859 | 2.4±3.4 | 1.8±1.3 | 2.8±3.8 | 0.847 |
| Days of hospitalization ^b^ | 9.8±7.7 | 5.8±1.8 | / | 0.04 |  | 9.8±7.4 | 5.8±2.3 | 0.001 |
| ICU admission, n(%) | 16 (80) | 0 | / | <0.001 | 41 (77.4) | 0 | / | <0.001 |
| Non-invasive ventilation, n(%) | 9 (45) | 0 | / | <0.001 | 18 (34.0) | 0 | / | <0.001 |
| Invasive ventilation, n(%) | 4 (20) | 0 | / | <0.001 | 14 (26.4) | 0 | / | <0.001 |
| PCIS ^b^ | 91.9±8.8 | 99.5±1.4 | / | <0.001 | 92.1±7.9 | 99.9±0.3 | / | <0.001 |
| Inflammation markers ^b^ | | | | |  |  |  |  |
| PCT, ng/mL | 0.95±2.0 | 0.53±1.1 | / | 0.519 | 1.8±6.2 | 0.3±0.5 | / | 0.189 |
| WBC, % | 11.3±6.8 | 8.4±3.8 | / | 0.135 | 9.5±4.0 | 10.7±4.0 | / | 0.175 |
| Neu, % | 0.5±0.2 | 0.3±0.2 | / | 0.019 | 0.5±0.2 | 0.4±0.2 | / | 0.045 |
| Lym, % | 0.4±0.2 | 0.6±0.2 | / | 0.016 | 0.4±0.2 | 0.5±0.2 | / | 0.009 |
| Mon, % | 0.08±0.05 | 0.07±0.03 | / | 0.403 | 0.08±0.04 | 0.07±0.02 | / | 0.091 |
| Other markers ^b^ | | | | | | | | |
| PT, s | 11.1±0.9 | 10.9±0.8 | / | 0.544 | 11.1±2.2 | 10.3±2.6 | / | 0.126 |
| INR | 1.0±0.1 | 0.9±0.1 | / | 0.530 | 0.9±0.2 | 0.9±0.2 | / | 0.277 |
| APTT, s | 32.8±5.7 | 30.3±7.6 | / | 0.311 | 30.2±4.6 | 27.3±8.4 | / | 0.036 |
| FIB, g/L | 3.2±1.4 | 2.9±1.1 | / | 0.568 | 2.7±1.1 | 3.7±2.6 | / | 0.017 |
| D-Dimer, mg/L | 1.0±1.9 | 3.6±8.7 | / | 0.286 | 1.2±2.0 | 0.7±1.2 | / | 0.194 |
| FDP, μg/mL | 3.4±4.5 | 10.5±23.4 | / | 0.281 | 3.3±3.6 | 2.6±2.7 | / | 0.302 |
| TT, s | 17.9±1.4 | 18.4±1.4 | / | 0.329 | 17.9±1.8 | 16.5±5.1 | / | 0.062 |

PCIS, pediatric critical illness score; PCT, procalcitonin; WBC, white blood cell; Neu %, neutrophil %; Lym %, [lymphocyte](https://fanyi.so.com/?src=onebox#lymphocyte)%; Mon %, monocytes; PT, prothrombin time; INR, international normalized ratio; APTT, activated partial thrombin time; FIB, fibrinogen; FIB, fibrinogen degradation product; TT, thrombin time. ^a^ P value among these groups; ^b^ Data are presented as the mean ± SD.
