# Supplementary figures and images for: Serum-integrated omics reveal the host response landscape for severe pediatric community-acquired pneumonia
Source: Crit Care. 2023 Mar 1;27:79. doi: 10.1186/s13054-023-04378-w (PMC9976684; doi:10.1186/s13054-023-04378-w)

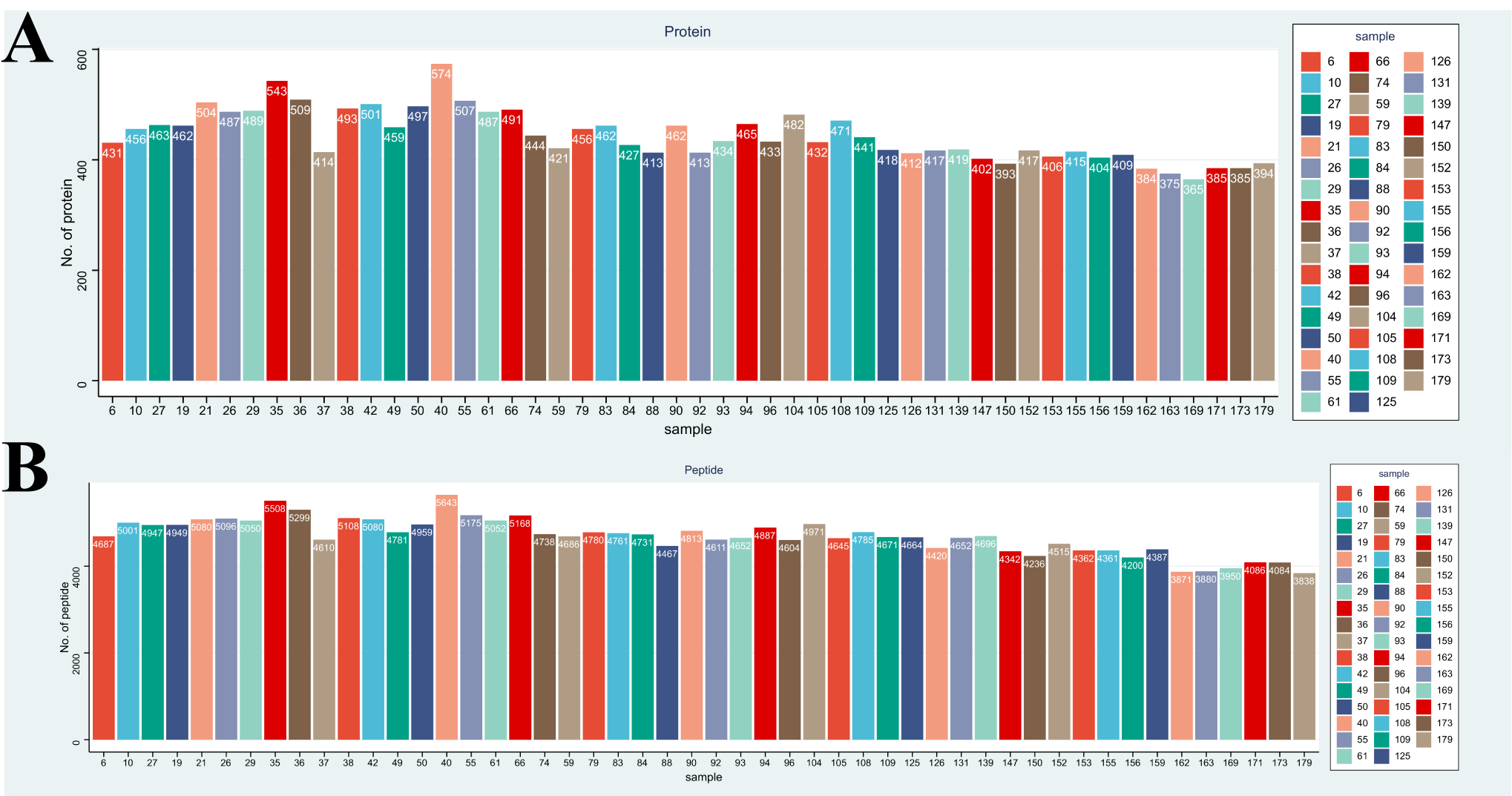

Supplement: Supplementary file 3 — Additional file 3. Fig. S1. Quality control and differentially abundant proteins (DAPs) in different samples. Distribution of the number of A quantified peptides and B proteins in the 50 serum samples from cohort 1. [file 13054_2023_4378_MOESM3_ESM.tif]

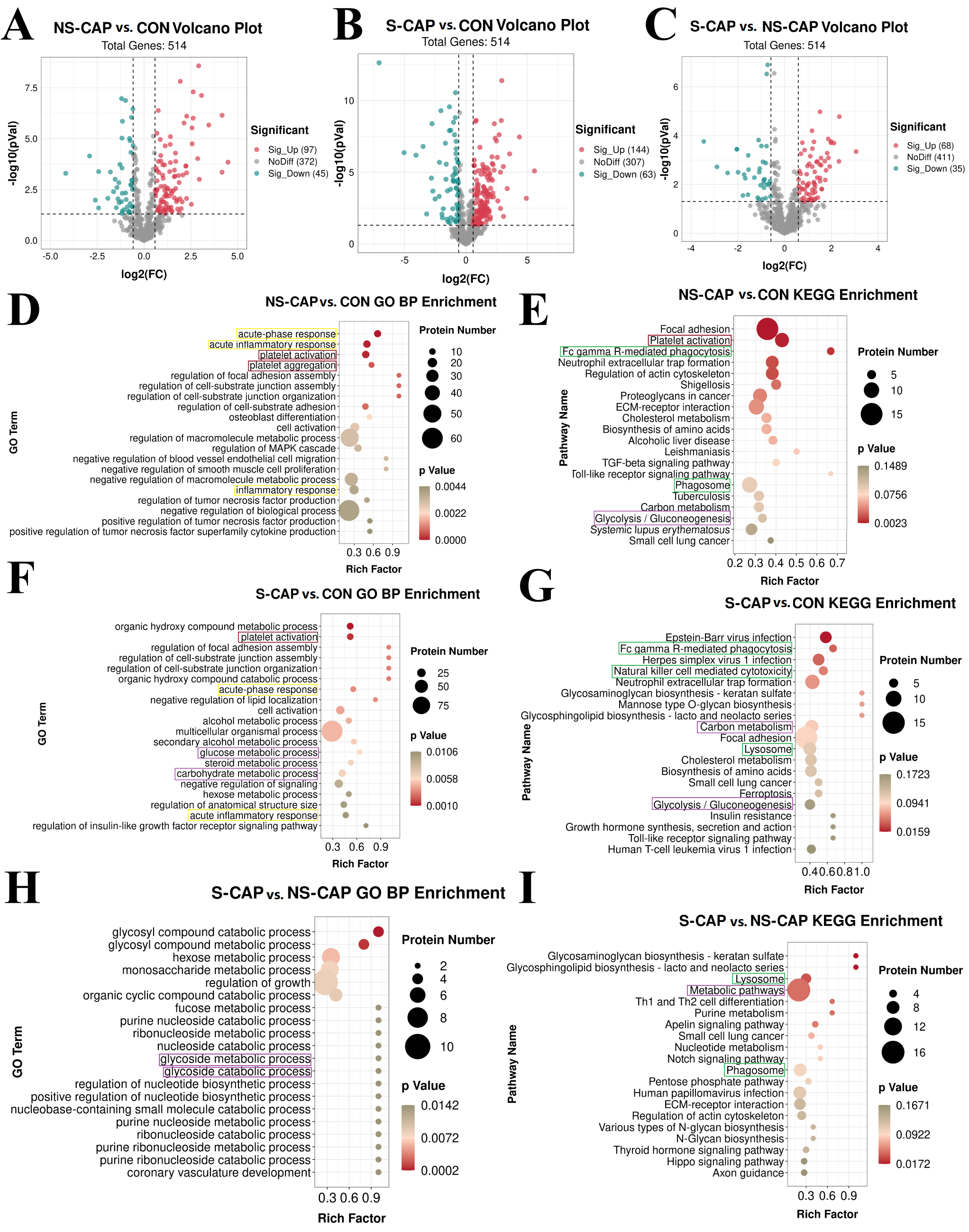

Supplement: Supplementary file 4 — Additional file 4. Fig. S2. DAPs in different pairwise comparison. Volcano plot comparing protein expression in A NS-CAP vs. CON, B S-CAP vs. CON and C S-CAP vs. NS-CAP. Proteins with FC >1.5 or <0.67 with P value <0.05 were considered to be significant DAPs. Number of significantly down- (green) and up- (red) regulated proteins are shown on top. GO-BP analysis of the DAPs from D NS-CAP vs. CON, F S-CAP vs. CON and H S-CAP vs. NS-CAP. KEGG analysis of the DAPs from E NS-CAP vs. CON, G S-CAP vs. CON and I S-CAP vs. NS-CAP. Top 20 terms are shown with red lines highlighting platelet-related pathways, yellow lines highlighting inflammatory-related pathways, green lines highlighting cell death-related pathways and purple lines highlighting metabolism-related pathways. [file 13054_2023_4378_MOESM4_ESM.tif]

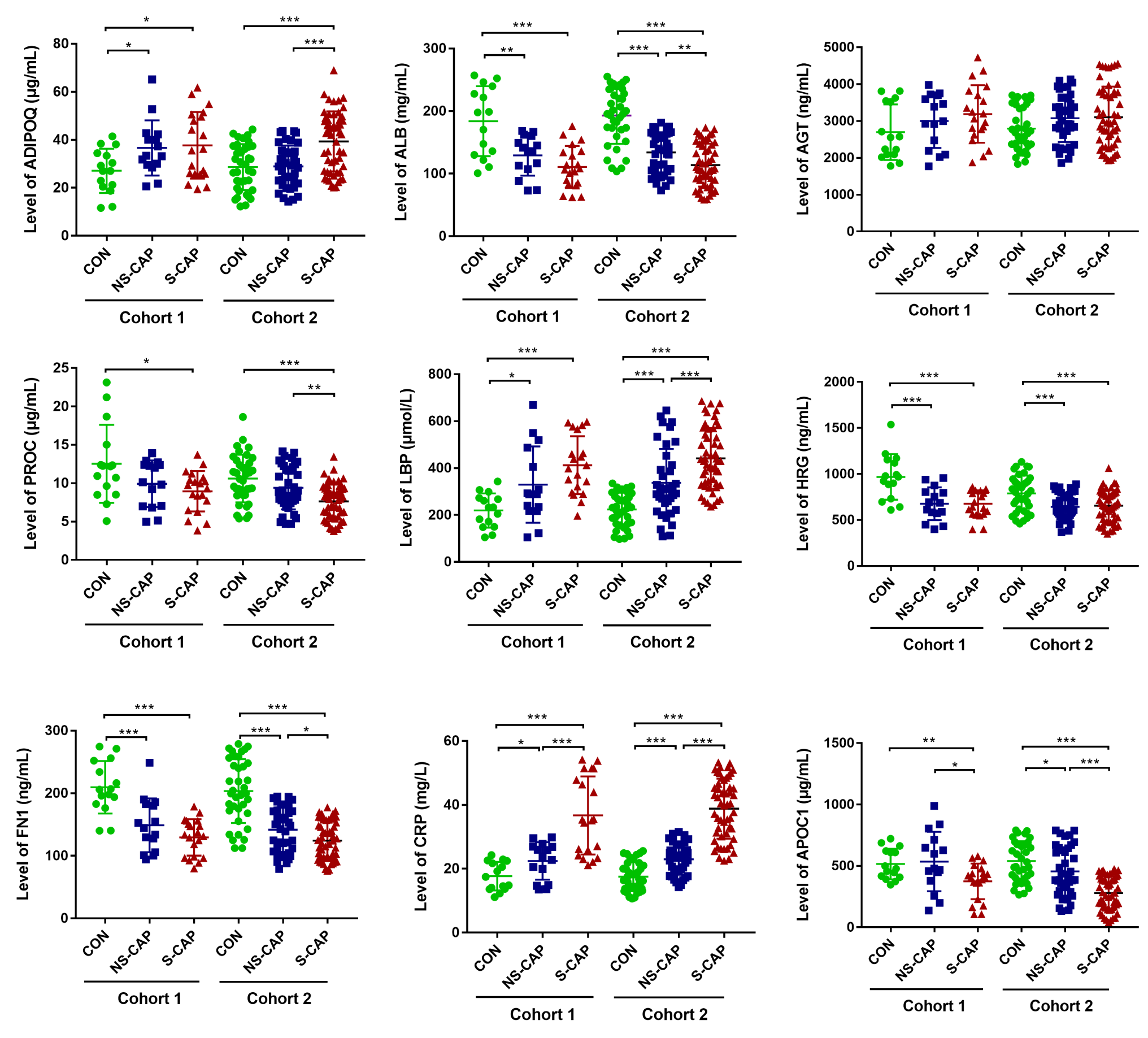

Supplement: Supplementary file 6 — Additional file 6. Fig. S3. Selected DAPs Verified using ELISA in Samples from Cohort 1 and Cohort 2. Protein levels for 9 selected DAPs were verified using ELISA. Statistical significance was determined by Student’s t test. *p < 0.05; **p < 0.01; ***p < 0.001. [file 13054_2023_4378_MOESM6_ESM.tif]

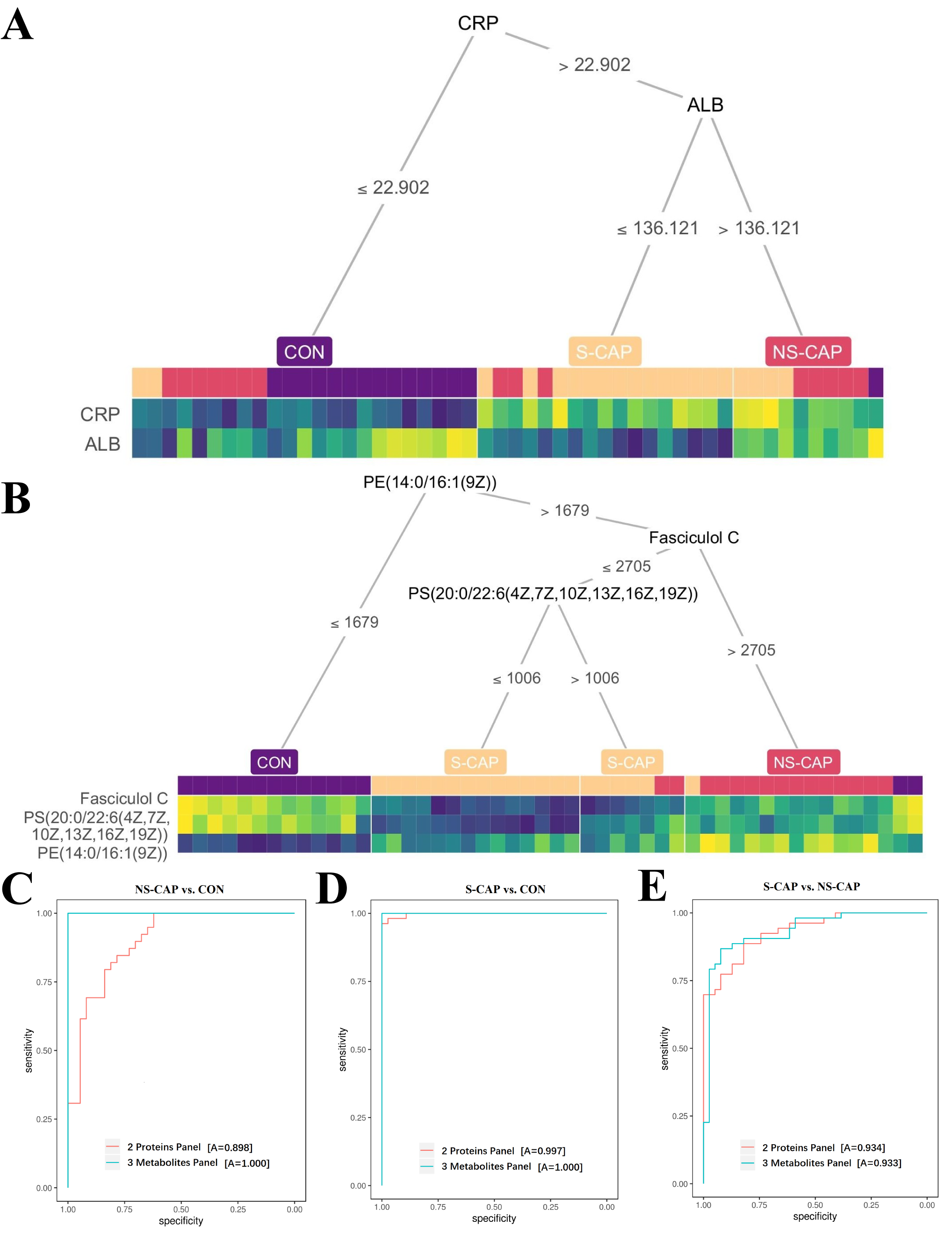

Supplement: Supplementary file 7 — Additional file 7. Fig. S4: Identification and verification of potential biomarker panels for classification of severe CAPs. A Classification and regression tree analysis using 2 DAPs with 2 terminal nodes. B Classification and regression tree analysis using 3 DAMs with 3 terminal nodes. C AUC values for the 2 combined panels were calculated to differentiate NS-CAPs from CONs in cohort 2. D AUC values for the 2 combined panels were calculated to differentiate S-CAPs from CONs in cohort 2. D AUC values for the 2 combined panels were calculated to differentiate S-CAPs from NS-CAPs in cohort 2. [file 13054_2023_4378_MOESM7_ESM.tif]

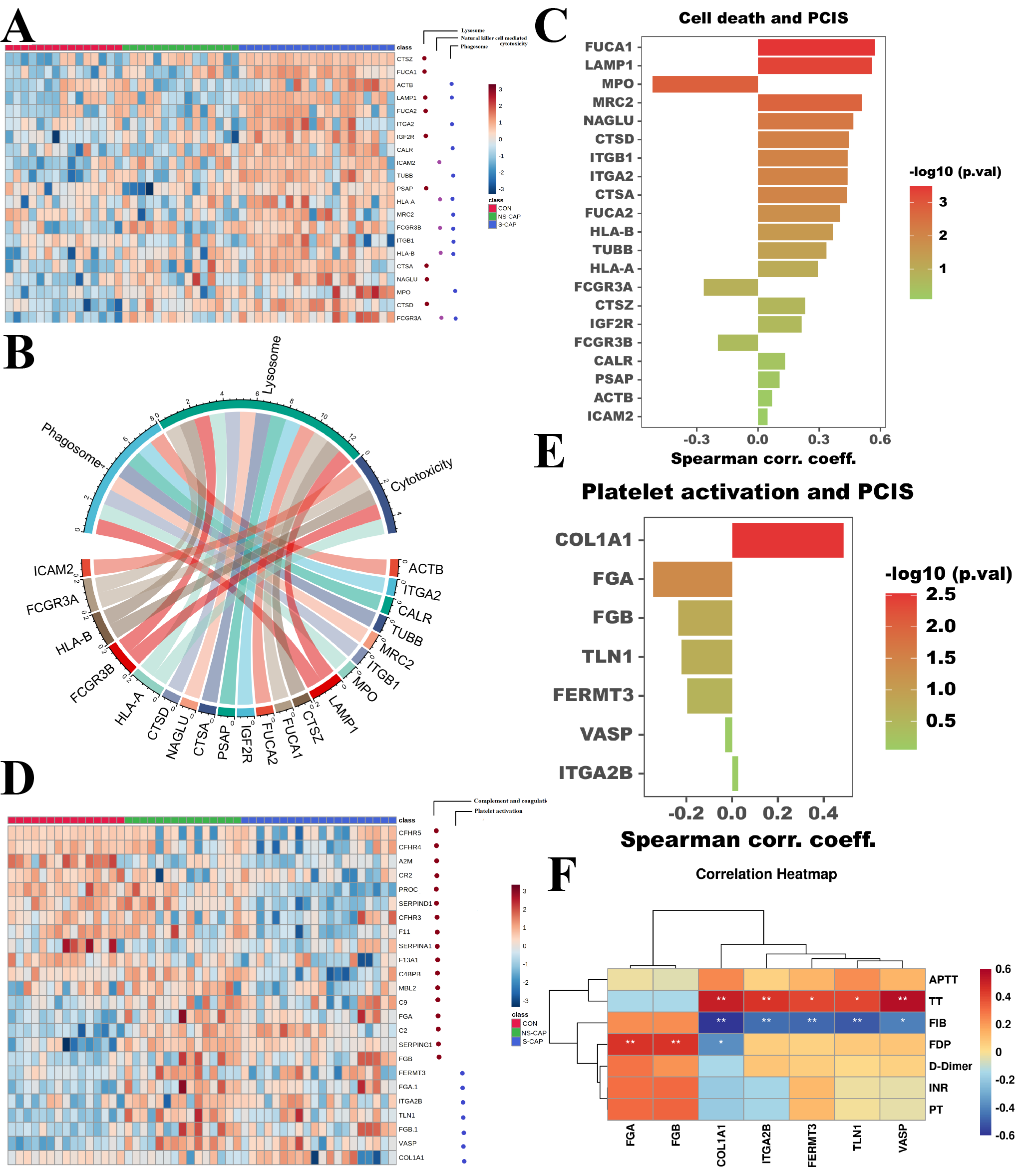

Supplement: Supplementary file 9 — Additional file 9. Fig. S5. Details of activated death system, dysregulated complement system and platelet function in S-CAP cases. A Heatmap showing expression levels of DAPs related to lysosome, nature killer cell mediated cytotoxicity, and phagosome. B The interaction network for proteins involved in the lysosome, nature killer cell mediated cytotoxicity, and phagosome pathways. C Correlation of death-related DAPs and pediatric critical illness score (PCIS). x axis depicts Spearman’s correlation coefficients. D Heatmap showing expression levels of proteins related to complement, coagulation cascades and platelet activation. E DAPs associated with the platelet activation pathway were correlated to PCIS. x axis shows Spearman’s correlation coefficients. F Spearman correlation heatmap between expression levels of DAPs associated with platelet-related pathways and clinical indices associated with platelet function. * means correlation p value < 0.05. ** means correlation p value < 0.01. Red means positive correlation. Blue means negative correlation. [file 13054_2023_4378_MOESM9_ESM.tif]

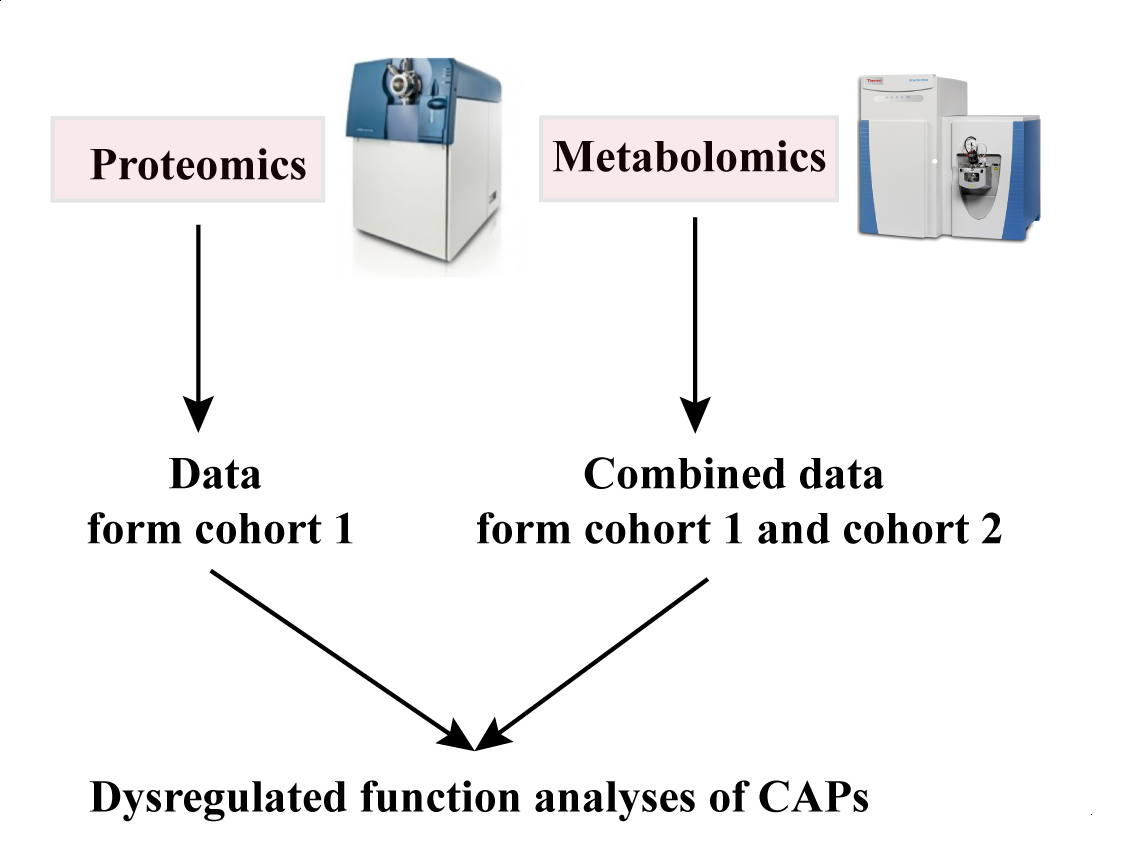

Supplement: Supplementary file 10 — Additional file 10. Fig. S6. Study overview of differentially abundant metabolites (DAMs) in different groups. Metabolomics data from cohort 1 and cohort 2 combined and analyzed. [file 13054_2023_4378_MOESM10_ESM.tif]

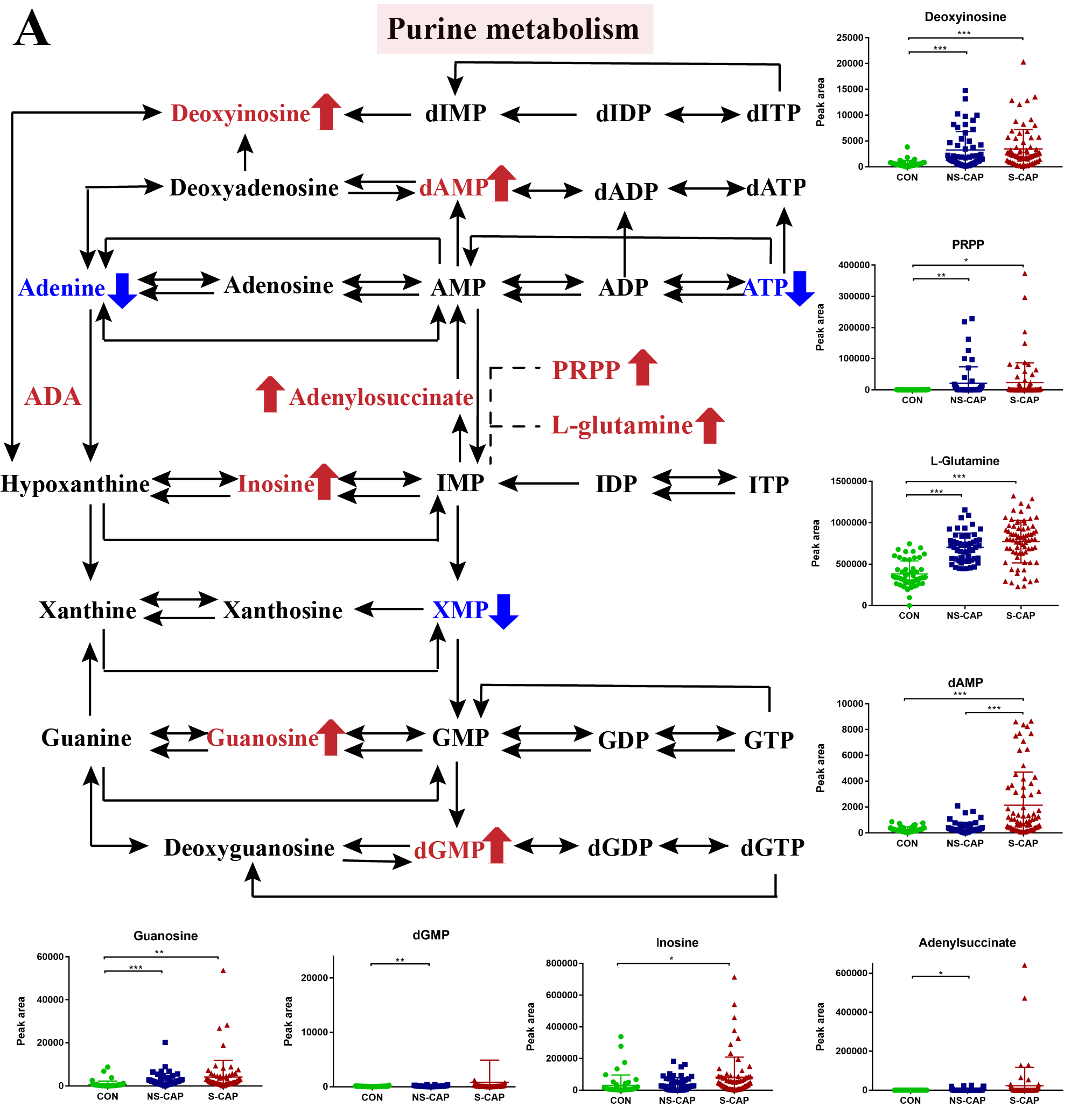

Supplement: Supplementary file 11 — Additional file 11. Fig. S7. Activation of Purine Metabolism in CAPs. Many purine metabolic intermediates (dAMP, dGMP, guanosine, deoxyinosine, and inosine) were significantly increased (as shown in red). Decreased proteins and metabolites were labeled in blue. Statistical significance was determined using the FDR-adjusted p-values. *p < 0.05; **p < 0.01; ***p < 0.001. [file 13054_2023_4378_MOESM11_ESM.tif]

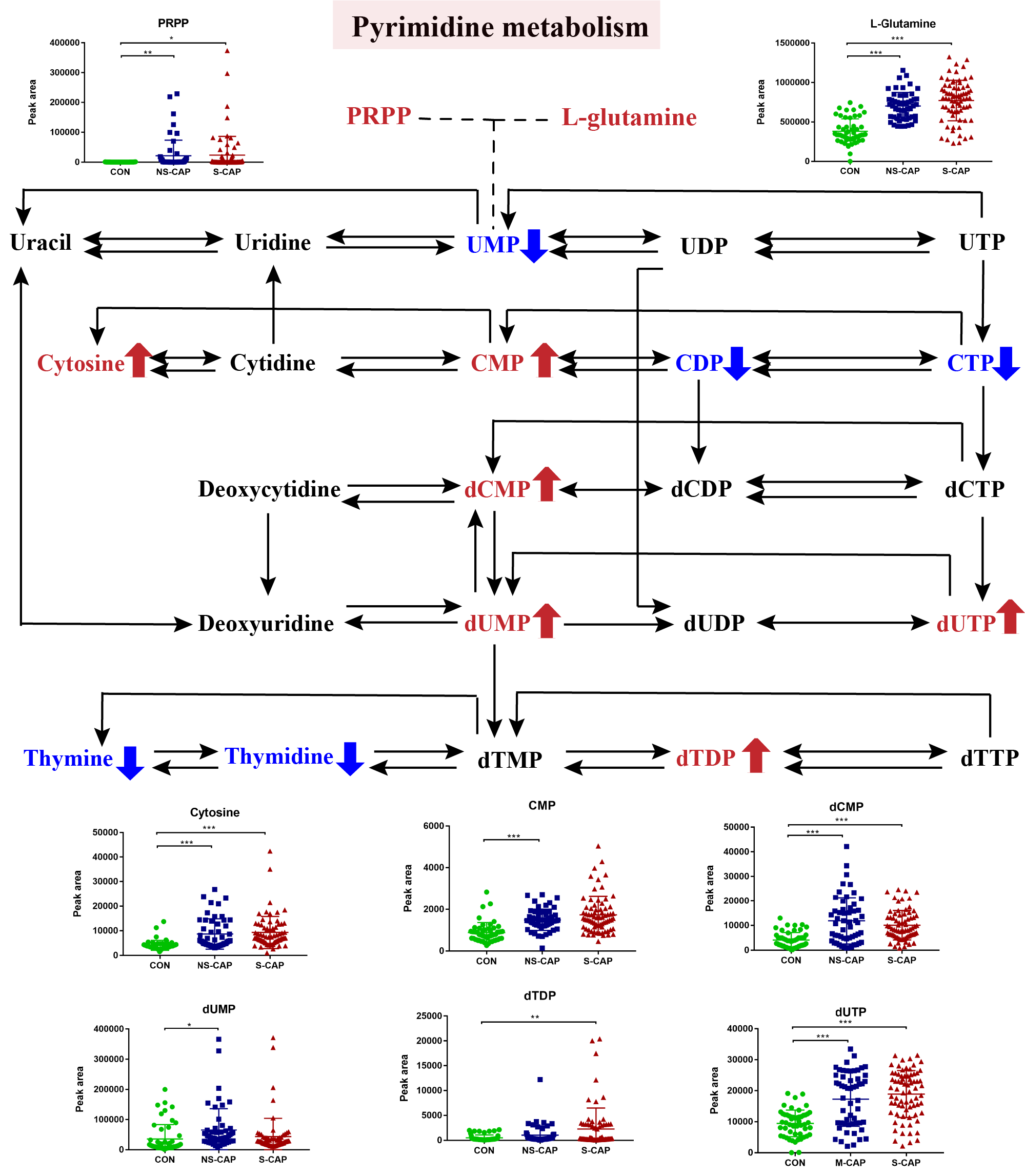

Supplement: Supplementary file 12 — Additional file 12. Fig. S8. Activation of pyrimidine metabolism in CAPs. Many pyrimidine metabolic intermediates (CMP, dCMP, dUMP, dUTP and dTDP were significantly increased (as shown in red). Decreased proteins and metabolites were labeled in blue. Statistical significance was determined using the FDR-adjusted p-values. *p < 0.05; **p < 0.01; ***p < 0.001. [file 13054_2023_4378_MOESM12_ESM.tif]
